# Delactylation of viral proteins by SIRT1 suppresses influenza A virus replication

**DOI:** 10.1128/mbio.02489-25

**Published:** 2026-03-18

**Authors:** Dongdong Chen, Gejie Zhao, Jia Zhou, Peng Sun, Shutong He, Changjie Lv, Yuhai Chen, Shouhai Zhu, Ming Gao, Guijie Guo

**Affiliations:** 1 Key Laboratory of Animal Pathogen Infection and Immunology of Fujian Province, College of Animal Sciences, Fujian Agriculture and Forestry University 12449, Fuzhou, China; 2 Fujian Province Joint Laboratory of Animal Pathogen Prevention and Control of the “Belt and Road”, College of Animal Sciences, Fujian Agriculture and Forestry University 12449, Fuzhou, China; 3 CAS Key Laboratory of Pathogenic Microbiology and Immunology, Institute of Microbiology, Chinese Academy of Sciences (CAS) 74519, Beijing, China; 4 Department of Oncology, Mayo Clinic 6915, Rochester, Minnesota, USA; 5 State Key Laboratory of Environmental Chemistry and Ecotoxicology, Research Center for Eco-Environmental Sciences, Chinese Academy of Sciences (CAS) 85415 , Beijing, China; 6 Engineering Research Center for Animal Breeding and Sustainable Production, College of Animal Sciences, Fujian Agriculture and Forestry University 12449, Fuzhou, China; Boston University Chobanian & Avedisian School of Medicine, Boston, Massachusetts, USA

**Keywords:** influenza A virus, SIRT1, vRNP, lactylation, virus replication

## Abstract

Lactylation, a protein post-translational modification driven by lactate, plays crucial roles in various physiological and pathological processes. However, the function and underlying mechanisms of lactylation in infection and pathogenesis of influenza A virus (IAV) remain poorly understood. Here, we demonstrated that sirtuin 1 (SIRT1) suppressed IAV replication by mediating delactylation of viral proteins. We found that IAV infection significantly downregulated the expression of SIRT1. Functional studies revealed that depletion of SIRT1 markedly enhanced replication of IAV, whereas overexpression of SIRT1 attenuated the viral replication. Mechanistically, we observed that SIRT1 interacted with IAV NP, PA, PB1, and PB2 proteins, components of the viral ribonucleoprotein (vRNP) complex, and restrained the activity of the vRNP complex, thereby repressing transcription and replication of the IAV genome. Furthermore, we identified that viral NP, PA, PB1, and PB2 proteins underwent lactylation during IAV infection, and SIRT1 facilitated the delactylation of NP, PA, and PB2. Loss of SIRT1 enhanced the lactylation of NP, PA, and PB2, while overexpression of SIRT1 impaired lactylation of these viral proteins during IAV infection. Moreover, inhibition or depletion of lactate dehydrogenase A (LDHA) robustly reduced the lactylation of NP, PA, and PB2, thereby abolishing the promotion of IAV replication caused by SIRT1 deficiency, suggesting that SIRT1 repressed IAV replication via delactylation of viral NP, PA, and PB2 proteins. Together, these results uncover a mechanism that SIRT1 suppresses IAV replication via delactylation of the vRNP components and elicit a critical role for the lactylation of viral proteins in regulating IAV infection and pathogenesis.

**IMPORTANCE:**

Lactylation plays vital roles in diverse pathological processes. However, the relationship between lactylation and influenza A virus (IAV) infection remains largely unexplored. Particularly, little information is available about the lactylation of viral proteins and their roles in the IAV pathogenesis. Here, viral proteins (NP, PA, PB1, and PB2) were identified to become lactylated during IAV infection. SIRT1 interacted with and promoted the delactylation of viral NP, PA, and PB2 proteins, thereby restraining the vRNP activity and inhibiting the viral transcription and replication. Interestingly, the expression of SIRT1 decreased following IAV infection, suggesting that IAV had evolved a mechanism to downregulate the SIRT1 expression, thereby ensuring appropriate lactylation of viral proteins and facilitating the IAV replication. Together, these findings reveal a critical role for the lactylation of viral proteins in regulating the replication of IAV and provide an important insight into the complicated interplay between the host and IAV.

## INTRODUCTION

Influenza A virus (IAV) is a zoonotic pathogen that can infect various species including humans, horses, pigs, poultry, dogs, cats, dairy cattle, and marine mammals, posing a serious threat to the animal and human health (1–6). IAV is a segmented, single-stranded, and negative-sense RNA virus, and the IAV genome consists of eight segments, including polymerase basic protein 2 (PB2), polymerase basic protein 1 (PB1), acidic polymerase (PA), hemagglutinin (HA), nucleoprotein (NP), neuraminidase (NA), matrix protein (M), and non-structural proteins (NS) (7–9). All eight viral RNA (vRNA) segments are associated with three polymerase subunits (PB2, PB1, and PA), and encapsidated by NP to form the viral ribonucleoprotein (vRNP) complex (6, 9–12). The transcription and replication of the IAV genome are carried out by the vRNP complex and occur in the nucleus of infected cells (11, 13). Additionally, the interaction between IAV vRNP complex and host factors is a critical determinant of the viral tropism and pathogenicity (14–16). Several host proteins, such as PLSCR1, BinCARD1, eEF1D, α-actinin-4, Hsp40, NF90, and MOV10, have been reported to be associated with the migration and function of the vRNP complex (17–23). However, the involvement and molecular mechanisms of lactylation in the regulation of the vRNP function remain unclarified.

Lactylation, a protein post-translational modification (PTM) driven by lactate, which is a key metabolite of the glycolytic pathway, has garnered widespread attention due to its crucial roles in a variety of physiological and pathological processes (24–27). Aerobic glycolysis is a hallmark of many viral infections, and emerging evidence suggests that lactylation plays vital roles in the viral pathogenesis and host antiviral immunity (28–36). Influenza virus infection triggers metabolic reprogramming in host cells, such as glycolysis and lactate production (37, 38). Inhibition of glycolysis suppresses influenza virus replication, while lactate facilitates the viral replication, implying that the influenza virus might exploit the glycolytic pathway to benefit its replication in the host (39). However, the relationship between lactylation and IAV infection remains largely unexplored. Particularly, little information is available about the lactylation of IAV proteins and their potential roles in the viral pathogenesis.

Lactylation is dynamically regulated by the writers that catalyze protein lactylation and erasers that remove the lactylation. Currently, the identified lactylation writers mainly include acyltransferases (GCN5, p300, CBP, TIP60, HBO1, and MOF) and alanyl-tRNA synthetases (AARS1 and AARS2), while the erasers primarily contain Zn^2+^-dependent histone deacetylases (HDAC1, HDAC2, and HDAC3) and NAD^+^-dependent sirtuins (SIRT1, SIRT2, and SIRT3) (40, 41). Of note, HDAC1, HDAC2, HDAC3, SIRT2, and SIRT3 have been reported to play important roles in the IAV infection (42–47). For instance, HDAC3 is critical for the antiviral immunity of macrophages and renders mice more resistant to IAV infections (42). Additionally, the SIRT2-HIF1α-dependent pathway is involved in the regulation of macrophage differentiation by PIEZO1-directed neutrophil extracellular traps (NETs) during influenza virus infection (48). Moreover, the expression of SIRT2 is downregulated in the influenza virus-infected cells, and treatment with the SIRT2 activator could restore the GSH production and NRF2 expression, thereby decreasing the replication of influenza virus (49). Similarly, SIRT3 is downregulated in the IAV-infected children’s serum samples and cell lines. Enhanced expression of SIRT3 impairs the replication of IAV and mitigates IAV-induced mitochondrial oxidative stress and inflammation (50). SIRT1 plays important roles in physiological processes including aging, metabolism, and autophagy and has also been linked to numerous pathological processes such as cancer, inflammatory and autoimmune diseases, owing to its capacity to deacetylate or delactylate histone and nonhistone substrates (51–55). Besides, SIRT1 has been reported to regulate immune and inflammatory responses following viral infections (56–59). However, the function and underlying mechanisms of SIRT1 in the infection and pathogenesis of IAV remain poorly understood.

In this study, we revealed SIRT1 as a negative regulator for IAV replication. Mechanistically, we found that viral NP, PA, PB1, and PB2 proteins underwent lactylation during IAV infection. Moreover, SIRT1 interacted with and mediated the delactylation of NP, PA, and PB2 proteins, thereby restraining the vRNP activity and inhibiting the transcription and replication of the IAV genome. Importantly, the promotion of IAV replication caused by SIRT1 knockdown was abolished by inhibition or depletion of LDHA that markedly decreased the lactylation of NP, PA, and PB2, suggesting that SIRT1 repressed IAV replication via delactylation of the viral NP, PA, and PB2 proteins. Together, these findings unveil a critical role of SIRT1 in suppressing IAV replication through modulating lactylation of the vRNP components and establish a role for the lactylation of viral proteins in regulating IAV infection and pathogenesis.

## MATERIALS AND METHODS

### Generation of SIRT1 knockdown and overexpression cell lines

The stable cell lines were generated using lentiviral expression systems as previously described (60). To generate stable cell lines, pLVX3-SIRT1, SIRT1 shRNA, and LDHA shRNA plasmids were constructed. The sequences of primers used for plasmid construction are listed in Table S1. Each of these plasmids, together with packaging plasmids psPAX2 and PMD2.G, was co-transfected into HEK293T cells by Lipo8000 (catalog no. C0533, Beyotime, China) according to the manufacturer’s instructions. After 48 h, the culture supernatant containing lentiviruses was harvested. A549 cells were digested, resuspended, and infected with the culture supernatant containing lentivirus. After 48 h, infected cells were selected by culturing in complete medium containing 2 µg/mL puromycin for an additional 48 h.

### Cell culture and viral infection

The human lung epithelial cells (A549), Madin-Darby canine kidney cells (MDCK), and human embryonic kidney 293T cells (HEK293T) were purchased from American Type Culture Collection (ATCC, Manassas, VA, USA). Influenza virus A/PR/8/34 (PR8), A/WSN/33 (WSN), A/Chicken/Fujian/MQ01/2015 (H9N2), and H3N2 were propagated in specific pathogen-free (SPF) embryonated chicken eggs as previously described (60). The cells were cultured in Dulbecco’s modified Eagle medium (DMEM) supplemented with 10% fetal bovine serum (FBS, Gibco, Indianapolis, IN, USA), 100 U/mL penicillin, and 100 μg/mL streptomycin at 37°C as previously described (61). For the viral infection, the cells were washed with phosphate-buffered saline (PBS) and infected with influenza viruses at the indicated multiplicity of infection (MOI). After 1 h of incubation at 37°C, the cells were washed with PBS and cultured in DMEM supplemented with 0.2 μg/mL TPCK-trypsin for the indicated duration.

### Cytotoxicity assay

Cytotoxicity assays were conducted using a Cell Counting Kit-8 (catalog no. GK10001, GlpBio, USA). A549 cells were seeded into 96-well culture plates and allowed to adhere. The cells were then incubated for 24 h with drugs at different concentrations. Following the treatment, 10 μL of CCK-8 solution was added to each well, and the plates were incubated at 37°C for 30 min. The absorbance at 450 nm was measured for each well using a microplate reader.

### Inhibitor and activator treatment

For the SIRT1 activator and inhibitor treatment, A549 cells were pretreated with the SIRT1 activator nicotinamide riboside (NR) (catalog no. HY-123033A, MCE, USA), along with or without the SIRT1 inhibitor selisistat (catalog no. HY-15452, MCE) for 1 h, and then infected with IAVs. For the LDHA inhibitor treatment, A549 cells were pretreated with the LDHA inhibitor oxamate (LDHi) (catalog no. HY-W013032A, MCE) for 1 h, and then infected with IAVs. The cells were collected for Western blot analysis, and the supernatants were harvested for TCID_50_ assays at 24 hpi (hour post-infection). Additionally, A549 cells were pretreated with the LDHA inhibitor oxamate for 1 h, followed by infection with IAV PR8 for 12 h. The cells were then treated with 20 μM chloroquine (CQ) (catalog no. C6628, Sigma, USA) or 10 μM MG132 (catalog no. M7449, Sigma) for an additional 10 h. The cells were collected for Western blot analysis.

### Western blot

Western blot was performed as described previously (62). Briefly, the cells were lysed in the NP-40 lysis buffer. Protein samples were separated by sodium dodecyl sulfate-polyacrylamide gel electrophoresis (SDS-PAGE), transferred to nitrocellulose (NC) membranes, and probed with specific antibodies. The following antibodies were used: Pan-Lac (1:1,000 dilution, catalog no. PTM-1401RM, PTM Biolab, Hangzhou, China), HA (1:10,000 dilution, catalog no. 81290-1-RR, Proteintech, USA), Flag (1:1,000 dilution, catalog no. A00170, GenScript, USA), Myc (1:1,000 dilution, catalog no. A00172, GenScript), NP (1:100,000 dilution, prepared in our laboratory), SIRT1 (1:1,000 dilution, catalog no. sc74465, Santa Cruz, USA), GAPDH (1:1,000 dilution, catalog no. TA-08, ZSGB-BIO, China), β-actin (1:1,000 dilution, catalog no. TA-09, ZSGB-BIO, China), PB1 (1:1,000 dilution, catalog no. GTX125923, GeneTex, USA), PB2 (1:2,000 dilution, catalog no. GTX125926, GeneTex), PA (1:1,000 dilution, catalog no. GTX118991, GeneTex), and LDHA (1:1,000 dilution, catalog no. 2012S, Cell Signaling Technology, USA).

### Reverse transcription and quantitative real-time PCR

Total RNA was extracted using TRIzol reagent (catalog no. DP424, TianGen Biotech, China) according to the manufacturer’s protocol. The RNA was reverse-transcribed into complementary DNA (cDNA) using a reverse transcription kit (catalog no. R312-02, Vazyme Biotech, China). Quantitative real-time PCR was performed using ChamQ Universal SYBR qPCR Master Mix (catalog no. Q312, Vazyme Biotech). For quantification, the 2^−ΔΔCt^ method was used to calculate relative RNA levels against 18S rRNA (60).

### TCID_50_ assay

Viral supernatants were harvested at the indicated time points post-infection and diluted serially onto MDCK cells cultured in 96-well plates (100 µL per well). The last column of the 96-well plate was used as a negative control containing 100 µL of cell maintenance medium. Cells were incubated at 37°C in a 5% CO_2_ incubator for 72 h. The TCID_50_ was determined by calculating log_10_ TCID_50_/mL on MDCK cells using the Reed-Muench method.

### Immunofluorescence assay

The cells were fixed at room temperature with 4% paraformaldehyde for 15 min, followed by permeabilization with 0.1% Triton X-100 for 5 min. After washing with PBS, the cells were blocked with 1% BSA-PBS at room temperature for 2 h. NP (1:20,000 dilution, catalog no. GTX14213, GeneTex) and SIRT1 (1:400 dilution, catalog no. 13161-1-AP, Proteintech) antibodies were incubated overnight at 4°C. Subsequently, slices were incubated with appropriate secondary antibodies Alexa Fluor 488-conjugated goat anti-mouse IgG (catalog no. A-11001, Invitrogen, USA), or Alexa Fluor 594-conjugated goat anti-rabbit IgG (catalog no. A-11012, Invitrogen) at 37°C for 1 h. After washing, the cells were stained with DAPI (catalog no. C1006, Beyotime) for 5 min to visualize the nuclei.

### Lactylome analysis

The lactylome analysis was performed by PTM Biolab. A549 cells were infected with IAV PR8, and the samples were collected at 24 hpi. Each group of samples was added to a lysis buffer (8 M urea, 1% protease inhibitor cocktail, 3 μM TSA, and 50 mM NAM) and lysed by sonication. Equal amounts of protein were taken for enzymatic digestion. The proteins were then reduced with 5 mM DTT at 37°C for 60 min, followed by alkylation with 11 mM IAA in the dark at room temperature for 45 min. The resulting peptides were desalted using a Strata X SPE column. The desalted peptides were dissolved in IP buffer (100 mM NaCl, 1 mM EDTA, 50 mM Tris-HCl, and 0.5% NP-40, pH 8.0) and incubated overnight at 4°C with pre-washed antibody resin (catalog no. PTM1404, PTM Biolab) under rotation. The resin was eluted three times with 0.1% trifluoroacetic acid. The eluates were then collected and lyophilized. The dried peptides were desalted according to the C18 ZipTips instructions, dried again under vacuum, and submitted for LC-MS/MS analysis. The DIA data were processed using Spectronaut (v.18) software, and searched against the Homo_sapiens_9606_SP_20231220.fasta concatenated with reverse decoy database, and IAV protein sequences.

### Immunoprecipitation (IP) assay

Immunoprecipitation assays were performed as described previously (63). The Protein A/G beads and Flag beads were pretreated before using. Protein A/G magnetic beads (catalog no. L-1004A, Biolinkedin, China) were washed three times with ice-cold PBS (5 min per time). Subsequently, 1 μg PA (catalog no. GTX118991, GeneTex), PB1 (catalog no. GTX125923, GeneTex), PB2 (catalog no. GTX125926, GeneTex), or NP (catalog no. GTX14213, GeneTex) antibodies were incubated with the beads overnight at 4°C. 1 µg IgG (catalog no. 2729 or 68860, Cell Signaling Technology) was used as a negative control. The next day, unbound antibodies were removed by washing with ice-cold PBS. Flag magnetic beads (catalog no. HY-K0207, MCE) were washed three times with ice-cold PBS before using.

The cells were collected, washed with PBS, and lysed in the IP lysis buffer (containing 1 mM PMSF) for 30 min. After centrifugation at 13,523 × *g* for 10 min, the supernatant was added to pretreated beads and incubated overnight at 4°C with gentle shaking. After discarding the supernatant, the beads were washed five times with the wash buffer (50 mM Tris-HCl, 200 mM NaCl, 2 mM EDTA, 1% Triton X-100, and 0.01% SDS). SDS-PAGE loading buffer was then added to the beads and boiled for 10 min to elute the bound proteins. The eluted proteins were subjected to Western blot analysis probed with the indicated antibodies.

### Dual-luciferase reporter assay

HEK293T cells were transfected with plasmids (pCAGGS-PA, pCAGGS-PB1, pCAGGS-PB2, and pCAGGS-NP), pPoll-Luc and Renilla luciferase expression plasmids along with Flag-SIRT1 or empty vector. The luciferase activity was measured using the dual-luciferase reporter assay system (catalog no. E1960, Promega, USA) and a Luminoskan Ascent luminometer (Thermo Scientific, USA) according to the manufacturer’s instructions. Relative luciferase activity was determined by the normalization of the firefly luciferase activity to that of Renilla luciferase. The luciferase reporter plasmids were kindly provided by Hongbo Zhou (College of Veterinary Medicine, Huazhong Agricultural University, Wuhan, China) and Jihui Ping (College of Veterinary Medicine, Nanjing Agricultural University, Nanjing, China).

### Quantification of viral RNA species

Control and SIRT1 overexpressing or knockdown A549 cells grown in six-well plates were infected with PR8 virus at an MOI of 10 for 4, 6, and 8 h, or at an MOI of 0.1 or 0.01 for 24 h. Total RNA was extracted. Relative quantities of viral NP genomic RNA (vRNA), complementary RNA (cRNA), and mRNA were determined by RT-qPCR as described previously (17, 64).

Control and SIRT1 overexpressing or knockdown HEK293T cells were transfected with PB2 (WT or E361A), PB1, PA, and NP, along with pPol I-NP-vRNA, pPol I-NA-vRNA, pPol I-NP-cRNA or pPol I-NA-cRNA plasmids. After 24 h, total RNA was extracted for RT-qPCR to detect the expression of NP and NA vRNA, cRNA, or mRNA as described previously (64, 65). The sequences of primers used for RT-qPCR are shown in Table S1.

### Statistical analysis

Data are represented as mean values ± SDs (standard deviations) from three independent experiments. Statistical analysis was performed by Student’s *t*-test using the GraphPad Prism 9.5.0 software (GraphPad Software Inc., San Diego, CA, USA). Differences were considered statistically significant when *P* values were less than 0.05. *P < 0.05, **P < 0.01, and ***P < 0.001.

## RESULTS

### Depletion of SIRT1 facilitates IAV replication

**Fig 1:**
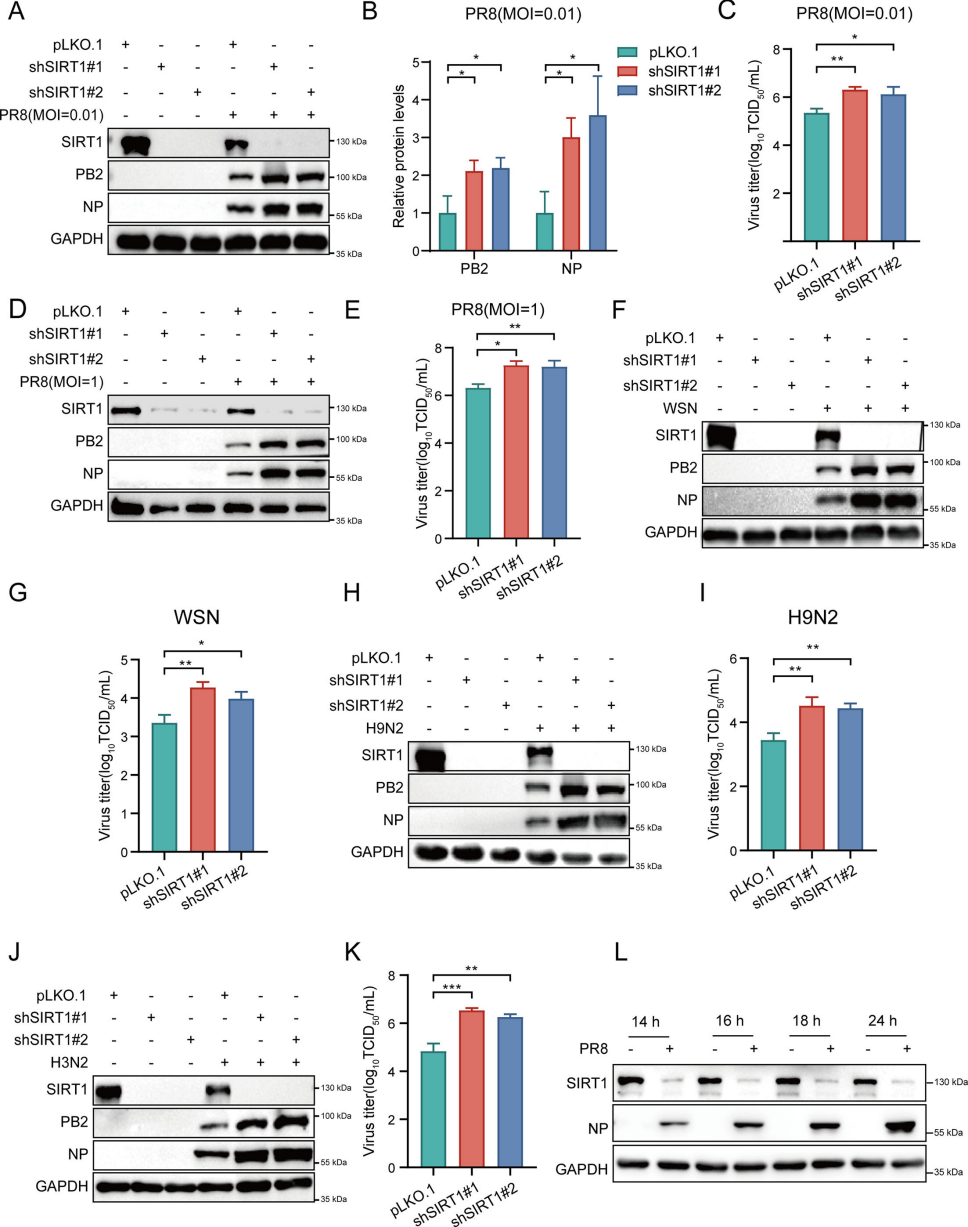
Depletion of SIRT1 promotes IAV replication. (**A–K**) Control and SIRT1 knockdown A549 cells were infected with influenza virus A/PR8/34 (H1N1) (MOI = 0.01) (**A–C**), A/PR8/34 (H1N1) (MOI = 1) (**D and E**), A/WSN/33 (H1N1) (MOI = 0.01) (**F and G**), H9N2 subtype virus (MOI = 0.01) (**H and I**), or H3N2 subtype virus (MOI = 0.01) (**J and K**) for 24 h. Viral NP and PB2 protein levels were examined by Western blot (**A, D, F, H, and J**). Viral titers in the supernatants were determined by TCID_50_ assays (**C, E, G, I, and K**). The band intensities of NP and PB2 proteins in panel **A** were analyzed by ImageJ software (**B**). (**L**) A549 cells were infected with influenza virus A/PR8/34 (H1N1) (MOI = 0.01). SIRT1 and viral NP protein levels were examined by Western blot at 14, 16, 18, and 24 hpi. Data are presented as mean ± SD of three independent experiments. *P < 0.05, **P < 0.01, and ***P < 0.001.

To define the role of SIRT1 in IAV infections, we generated A549 cells stably expressing specific shRNAs targeting SIRT1 (sh-SIRT1#1 and sh-SIRT1#2) or control (pLKO.1) using lentiviral vectors. The protein levels of SIRT1 were dramatically reduced in A549 cells expressing SIRT1 shRNAs compared with those in control cells (Fig. 1A). Then, control and SIRT1 knockdown cells were infected with influenza virus A/PR8/34 (H1N1) (MOI = 0.01, 0.1, or 1), and viral loads were determined by Western blot and TCID_50_ assays. Notably, the levels of viral NP and PB2 proteins were dramatically increased in SIRT1 knockdown cells compared with those in control cells following PR8 virus infection (Fig. 1A, B and D; Fig. S1A). In line with this, SIRT1 knockdown significantly increased IAV titers in the cells compared with the control (Fig. 1C and E; Fig. S1B). Furthermore, we infected control and SIRT1 knockdown cells with other strains of IAV including A/WSN/33 (H1N1), H9N2, and H3N2 subtype virus, and consistently, deprivation of SIRT1 enhanced the replication of the viruses in the cells (Fig. 1F through K; Fig. S1C through E). These results indicate that depletion of SIRT1 promotes the replication of IAV.

Next, we determined the expression of SIRT1 during IAV infection. A549 cells were infected with influenza virus A/PR8/34 (H1N1), and SIRT1 protein levels were detected by Western blot. As shown in Fig. 1L, a significant decrease in the SIRT1 protein levels was observed in the cells following PR8 virus infection. To further address whether the expression of SIRT1 can also be regulated by different strains of IAV, we utilized H3N2 and H9N2 IAV strains. Consistently, H3N2 or H9N2 infection was found to significantly downregulate SIRT1 expression (Fig. S1F and G). Then, we overexpressed Flag-tagged IAV proteins (PA, PB1, PB2, NP, HA, NA, NS1, or M2) in HEK293T cells and examined the SIRT1 expression. We observed that overexpression of PA, but not other viral proteins, led to a decrease in the SIRT1 protein levels (Fig. S1H), implying that PA may be involved in the downregulation of SIRT1 by IAV infection. Together, these results suggest that SIRT1 is downregulated by infection with various strains of IAV, and depletion of SIRT1 facilitates the replication of IAV.

### Overexpression of SIRT1 inhibits IAV replication

**Fig 2:**
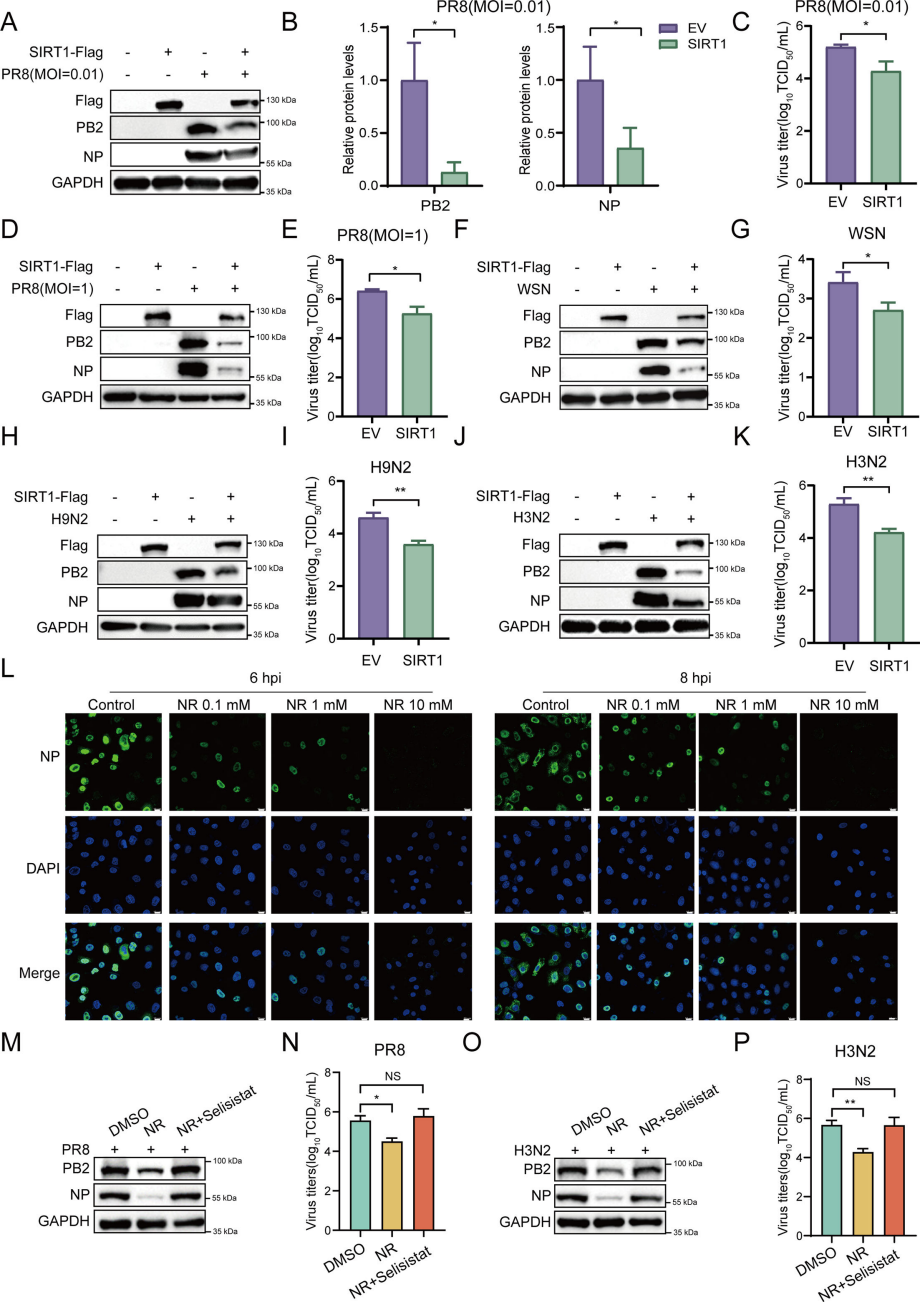
Overexpression of SIRT1 attenuates IAV replication. (**A–K**) Control and SIRT1 overexpressing A549 cells were infected with influenza virus A/PR8/34 (H1N1) (MOI = 0.01) (**A–C**), A/PR8/34 (H1N1) (MOI = 1) (**D and E**), A/WSN/33 (H1N1) (MOI = 0.01) (**F and G**), H9N2 subtype virus (MOI = 0.01) (**H and I**), or H3N2 subtype virus (MOI = 0.01) (**J and K**) for 24 h. Viral NP and PB2 protein levels were detected by Western blot (**A, D, F, H, and J**). Viral titers in the supernatants were determined by TCID_50_ assays (**C, E, G, I, and K**). The band intensities of NP and PB2 proteins in panel **A** were analyzed by ImageJ software (**B**). (**L**) A549 cells were pretreated with nicotinamide riboside (NR) (0.1 mM, 1 mM, or 10 mM) for 1 h, followed by infection with PR8 virus (MOI = 5). At 6 and 8 hpi, the cells were fixed and stained with anti-NP antibody, followed by incubation with the secondary antibody (Alexa Fluor 488, green). The nuclei were stained with DAPI. Scale bar, 10 μm. (**M–P**) A549 cells were pretreated with NR (1 mM) and Selisistat (1 μM) for 1 h, followed by infection with PR8 (**M and N**) or H3N2 (**O and P**) virus (MOI = 0.01). Viral NP and PB2 protein levels were detected by Western blot at 24 hpi (**M and O**). Viral titers in the supernatants were determined by TCID_50_ assays (**N and P**). Data are presented as means ± SDs of three independent experiments. *P < 0.05 and **P < 0.01.

On the other hand, we also evaluated the effect of SIRT1 overexpression on the replication of IAV. A549 cells stably expressing SIRT1 or empty vector (EV) were generated using lentiviral vectors. These cells were then infected with influenza virus A/PR8/34 (H1N1), and replication of the virus was examined. As expected, decreased IAV titers and reduced viral NP and PB2 protein levels were observed in SIRT1 overexpressing A549 cells compared with those in control cells after PR8 virus infection (Fig. 2A through E; Fig. S2A and B). Similarly, overexpression of SIRT1 led to a significant decrease in IAV titers and viral protein levels compared with the control cells, in response to infection with influenza virus A/WSN/33 (H1N1), H9N2, and H3N2 subtype viruses (Fig. 2F through K; Fig. S2C through E). In addition, we also evaluated the functional involvement of the activation of SIRT1 in the regulation of IAV replication by SIRT1. We treated cells with the SIRT1 agonist nicotinamide riboside (NR) (66), and examined IAV replication by Western blot and immunofluorescence assays. Significantly decreased levels of viral PB2 and NP proteins were observed in the cells treated with the SIRT1 agonist NR, compared with those in untreated cells following IAV infection (Fig. 2L; Fig. S2F through K), indicating that the SIRT1 agonist impaired IAV replication in the cells. Moreover, we observed that treatment with the SIRT1 inhibitor selisistat (67) could rescue the downregulation of IAV replication caused by the NR treatment (Fig. 2M through P; Fig. S2L through Q). Together, these observations suggest that altering SIRT1 expression and activity has profound effects on the replication of IAV.

### SIRT1 interacts with IAV NP, PA, PB1, and PB2 proteins

**Fig 3:**
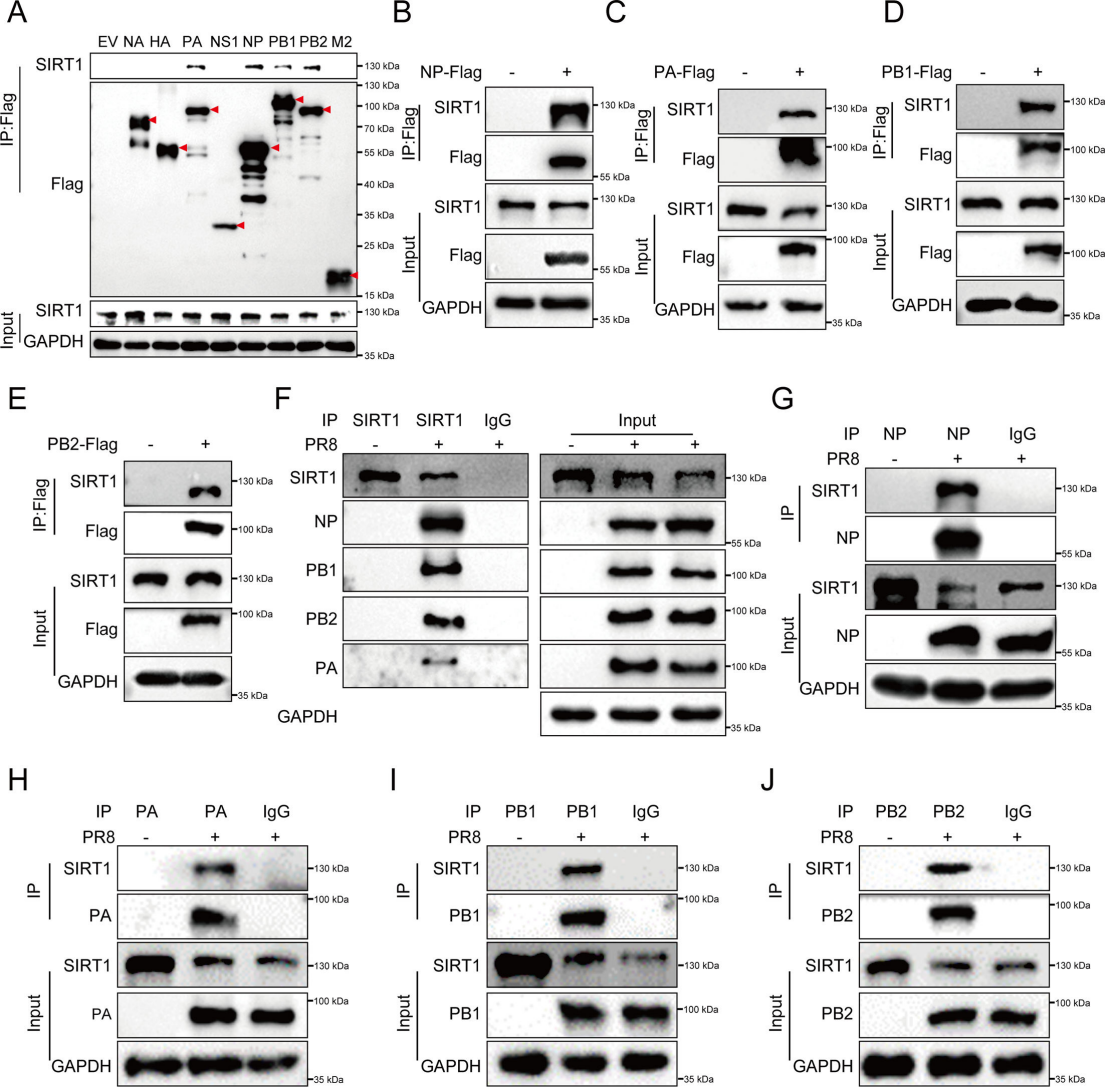
SIRT1 interacts with IAV NP, PA, PB1, and PB2 proteins. (**A**) HEK293T cells were transfected with Flag-tagged NA, HA, PA, NS1, NP, PB1, PB2, M2, or EV for 24 h, followed by IP assays using anti-Flag antibody and Western blot probed with anti-SIRT1 antibody. (**B–E**) Flag-tagged NP (**B**), PA (**C**), PB1 (**D**), or PB2 (**E**) was transfected into HEK293T cells, and IP assays were performed using anti-Flag antibody, followed by Western blot to detect SIRT1 using anti-SIRT1 antibody. (**F**) A549 cells were infected with influenza virus A/PR8/34 (H1N1) (MOI = 0.1), followed by IP assays using anti-SIRT1 antibody with IgG as a negative control, and Western blot assays using anti-NP, anti-PB1, anti-PB2, or anti-PA antibody. (**G–J**) A549 cells were infected with influenza virus A/PR8/34 (H1N1) (MOI = 0.1), and IP assays were performed using anti-NP (**G**), anti-PA (**H**), anti-PB1 (I), or anti-PB2 (**J**) antibody, with IgG as a negative control, followed by Western blot using anti-SIRT1 antibody.

Next, we sought to dissect the molecular mechanisms by which SIRT1 represses IAV replication. For this, we overexpressed Flag-tagged IAV proteins in HEK293T cells and performed immunoprecipitation (IP) assays, followed by Western blot probed with the SIRT1 antibody to screen for potential viral proteins that interacted with SIRT1. Among these viral proteins, IP of NP, PA, PB1, or PB2, components of the vRNP complex, effectively brought down SIRT1 in the whole-cell lysate (Fig. 3A), implying an interaction between SIRT1 and the IAV vRNP complex. To validate this, Flag-tagged NP, PA, PB1, or PB2 was transfected into HEK293T cells, respectively, followed by IP assays. As expected, IP of Flag-NP, PA, PB1, or PB2 clearly brought down SIRT1 in the whole-cell lysate (Fig. 3B through E).

To further confirm the interaction between SIRT1 and the vRNP complex, we examined the association of endogenous SIRT1 with NP, PA, PB1, and PB2 during IAV infection. A549 cells were infected with influenza virus A/PR8/34 (H1N1) and subjected to IP assays using anti-SIRT1 antibody with IgG as a negative control, followed by Western blot to detect NP, PA, PB1, or PB2 in the whole-cell lysate. As shown in Fig. 3F, IP of endogenous SIRT1 effectively pulled down NP, PA, PB1, and PB2 in the PR8 virus-infected cell lysate. Reciprocally, A549 cells were infected with influenza virus A/PR8/34 (H1N1), and IP assays were performed using anti-NP, PA, PB1, or PB2 antibody, followed by Western blot to detect endogenous SIRT1 in the whole-cell lysate. We found that NP, PA, PB1, or PB2 Co-IPed with endogenous SIRT1 in the PR8 virus-infected cell lysate (Fig. 3G through J). In addition, immunofluorescence data showed that SIRT1 exhibited colocalization with NP during IAV infection (Fig. S3). Together, these results suggest that SIRT1 interacts with NP, PA, PB1, and PB2, components of the vRNP complex, during IAV infection.

### SIRT1 inhibits viral transcription and replication

**Fig 4:**
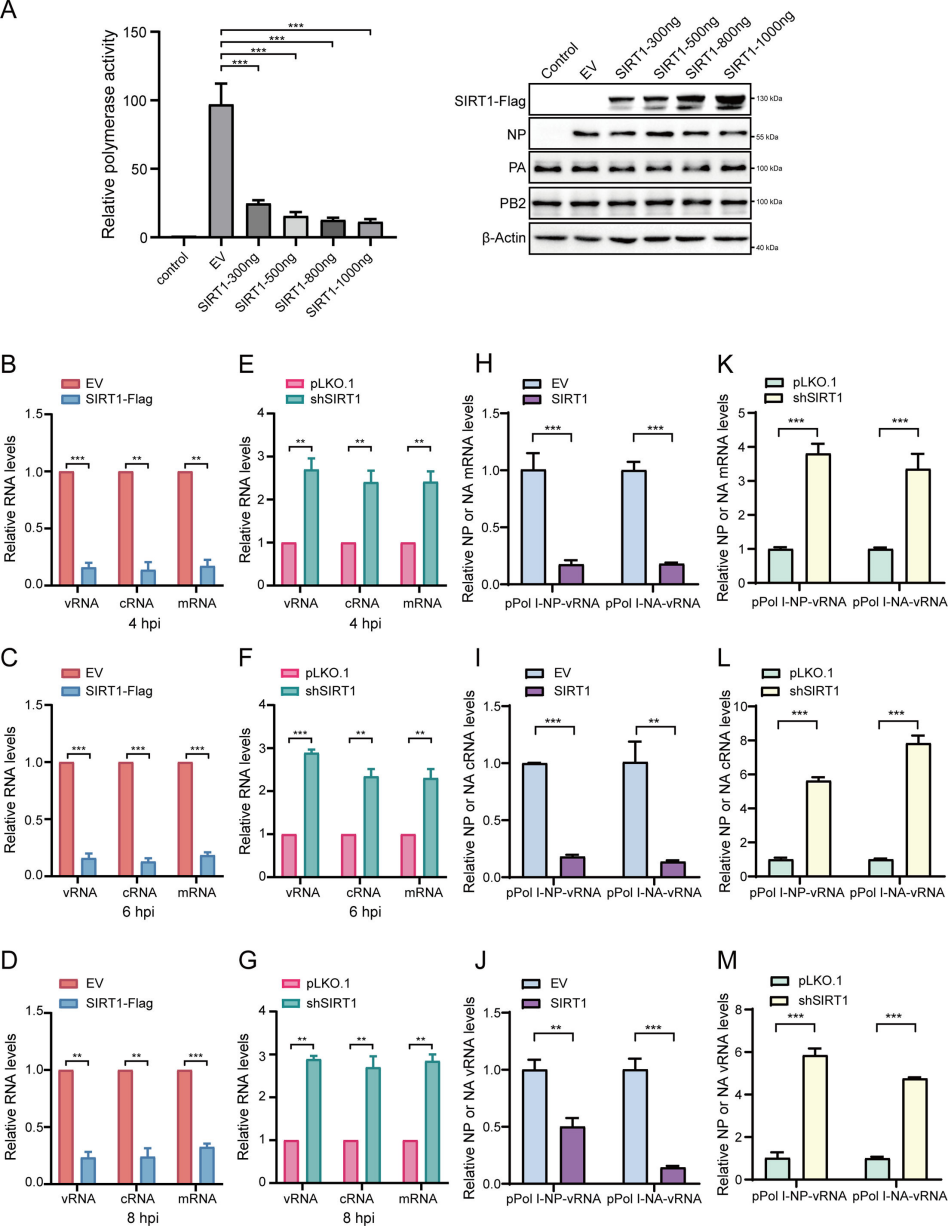
SIRT1 inhibits influenza virus transcription and replication. (**A**) HEK293T cells were transfected with plasmids (pCAGGS-PA, pCAGGS-PB1, pCAGGS-PB2, and pCAGGS-NP), pPoll-Luc and Renilla luciferase expression plasmids, along with increasing amounts of Flag-SIRT1 or EV. The luciferase activity was measured at 24 h after the transfection. (**B–D**) Control and SIRT1 overexpressing A549 cells were infected with PR8 virus at an MOI of 10. Total RNA was collected at 4 hpi (**B**), 6 hpi (**C**), and 8 hpi (**D**). NP vRNA, cRNA, and mRNA levels were analyzed by RT-qPCR. (**E–G**) Control and SIRT1 knockdown A549 cells were infected with PR8 virus at an MOI of 10. Total RNA was collected at 4 hpi (**E**), 6 hpi (**F**), and 8 hpi (**G**). NP vRNA, cRNA, and mRNA levels were analyzed by RT-qPCR. (**H–J**) HEK293T cells were transfected with either pPol I-NP-vRNA or pPol I-NA-vRNA, along with plasmids (pCAGGS-PA, pCAGGS-PB1, pCAGGS-PB2, pCAGGS-NP, and Flag-SIRT1 or EV). The levels of NP and NA mRNA, cRNA, and vRNA were measured at 24 h after the transfection. (**K–M**) Control and SIRT1 knockdown HEK293T cells were transfected with either pPol I-NP-vRNA or pPol I-NA-vRNA, along with plasmids (pCAGGS-PA, pCAGGS-PB1, pCAGGS-PB2, and pCAGGS-NP). The levels of NP and NA mRNA, cRNA, and vRNA were detected at 24 h after the transfection. Data are shown as means ± SDs (*n* = 3). **P < 0.01 and ***P < 0.001.

A key feature of the IAV life cycle is that transcription and replication of the viral genome occur in the nucleus of infected cells that are carried out by the vRNP complex. This prompted us to ask whether SIRT1 might interfere with the vRNP activity via interaction with components of the IAV vRNP complex, thereby suppressing the viral replication. To test this, HEK293T cells were transfected with expression constructs of the vRNP complex proteins (PB2, PB1, PA, and NP), and a reporter plasmid containing the terminal coding and noncoding sequences from the NS segment and the luciferase gene driven by the human RNA polymerase I promoter and terminator, along with EV or SIRT1 plasmid, followed by detection of the luciferase activity of the cell lysates. As shown in Fig. 4A, a significant decrease in the vRNP activity was observed in the cells overexpressing SIRT1 compared with that in EV control cells, suggesting that SIRT1 inhibited the vRNP activity, which may result in the repression of transcription and replication of the IAV genome.

To further delineate the effect of SIRT1 overexpression on the viral transcription and replication of IAV, we infected control and SIRT1 overexpressing A549 cells with PR8 virus at an MOI of 10. The vRNA, mRNA, and cRNA levels of NP were then measured by RT-qPCR at 4, 6, and 8 hpi, respectively. We found that the levels of all three species of viral RNA were significantly decreased in SIRT1 overexpressing cells compared with those in control cells following PR8 virus infection (Fig. 4B through D). In addition, we infected control and SIRT1 overexpressing A549 cells with PR8 virus at an MOI of 0.01 or 0.1, and the NP vRNA, mRNA, and cRNA levels were measured at 24 hpi. The levels of all three species of viral RNA were significantly reduced in SIRT1 overexpressing cells compared with control cells following PR8 virus infection (Fig. S4A and B). These data indicate that overexpression of SIRT1 suppresses transcription and replication of the IAV genome. On the other hand, we also evaluated the effect of SIRT1 knockdown on the viral transcription and replication. Control and SIRT1 knockdown A549 cells were infected with PR8 virus at an MOI of 10 for 4, 6, and 8 h, or at an MOI of 0.01 or 0.1 for 24 h. The vRNA, mRNA, and cRNA levels of NP were detected by RT-qPCR, respectively. As shown in Fig. 4E through G and Fig. S4C and D, depletion of SIRT1 led to a significant increase in the levels of all three species of viral RNA.

To further address the effect of SIRT1 on the viral transcription and replication of IAV, control and SIRT1 overexpressing HEK293T cells were transfected with PB2, PB1, PA, and NP expression plasmids, along with pPol I-NA-vRNA or pPol I-NP-vRNA. After 24 h, total RNA was extracted for RT-qPCR to detect the expression of NA or NP vRNA, cRNA, and mRNA. We observed that overexpression of SIRT1 significantly decreased the expression of vRNA, as well as the cRNA and mRNA replicated or transcribed from the vRNA (Fig. 4H through J). In contrast, depletion of SIRT1 led to a significant increase in the expression of vRNA, cRNA, and mRNA (Fig. 4K through M).

Additionally, we performed another experiment, in which a replication-competent but transcription-deficient PB2 (PB2-E361A) was employed (Fig. S4E and H). Control and SIRT1 overexpressing HEK293T cells were transfected with PB2-E361A, PB1, PA, and NP expression plasmids, along with pPol I-NA-vRNA or pPol I-NP-vRNA. As shown in Fig. S4F and G, I and J, overexpression of SIRT1 significantly decreased the expression of vRNA and cRNA of NP or NA, while loss of SIRT1 led to a significant increase in the expression of vRNA and cRNA (Fig. S4K through N). Moreover, control and SIRT1 overexpressing HEK293T cells were transfected with PB2-E361A, PB1, PA, and NP expression plasmids, along with pPol I-NA-cRNA or pPol I-NP-cRNA. Our data showed that overexpression of SIRT1 significantly reduced the expression of NP or NA vRNA (Fig. S4O), while knockdown of SIRT1 led to a significant increase in the vRNA expression (Fig. S4P). Together, these results suggest that SIRT1 interacts with the vRNP complex and interferes with the vRNP activity, thereby limiting the transcription and replication of the IAV genome.

### Viral NP, PA, PB1, and PB2 proteins undergo lactylation during IAV infection

**Fig 5:**
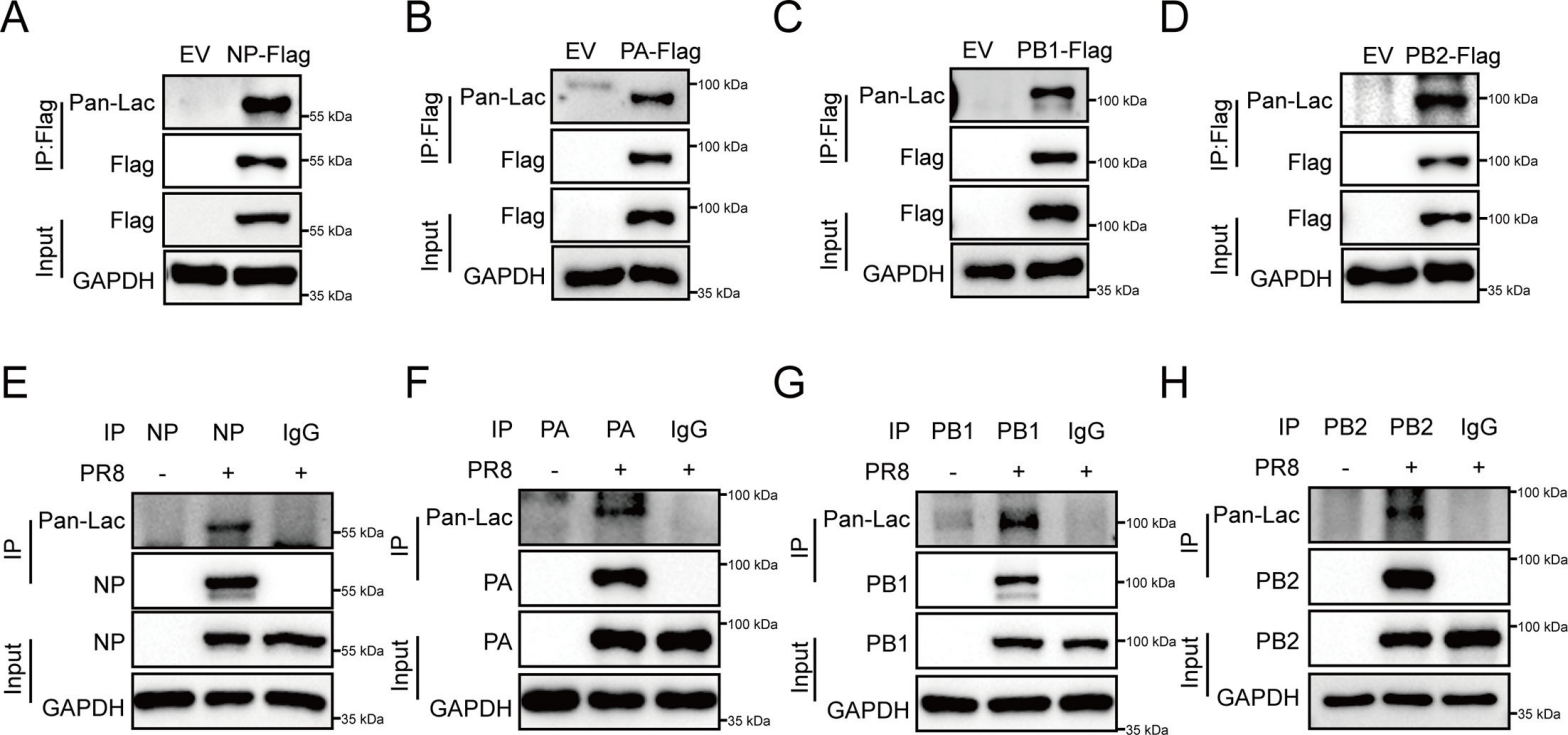
Viral NP, PA, PB1, and PB2 proteins undergo lactylation during IAV infection. (**A–D**) Flag-tagged NP (**A**), PA (**B**), PB1 (**C**) or PB2 (**D**), was transfected into HEK293T cells, and IP assays were performed using anti-Flag antibody, followed by Western blot analysis to detect lactylation of these viral proteins using anti-pan lac antibody. (**E–H**) A549 cells were infected with influenza virus A/PR8/34 (H1N1) (MOI = 0.1), and IP assays were performed using anti-NP (**E**), anti-PA (**F**), anti-PB1 (**G**), or anti-PB2 (**H**) antibody, with IgG as a negative control, followed by Western blot analysis using anti-pan lac antibody.

Lactylation plays important roles in a variety of pathological processes, including viral infections (68, 69). However, little information is available about lactylation of IAV proteins and their potential roles in the viral pathogenesis. To test this, we overexpressed Flag-tagged NP, PA, PB1, or PB2 in HEK293T cells, respectively, and performed IP assays, followed by Western blot probed with the anti-lactyl lysine antibody to evaluate lactylation of these viral proteins. Notably, a clear lactylation signal was observed in the Flag-NP, PA, PB1, or PB2 immunoprecipitate (Fig. 5A through D), implying the occurrence of lactylation in NP, PA, PB1, and PB2 proteins. To further confirm this, we examined the lactylation of these viral proteins under the circumstance of IAV infection. A549 cells were infected with influenza virus A/PR8/34 (H1N1), and IP assays were performed using anti-NP, PA, PB1, or PB2 antibody with IgG as a negative control, followed by Western blot to detect the lactylation of NP, PA, PB1, or PB2 in the cell lysates. As shown in Fig. 5E through H, NP, PA, PB1, and PB2 were clearly lactylated following PR8 virus infection.

Furthermore, we performed a lactylome analysis to profile the lactylation of viral proteins and differentially lactylated host proteins in A549 cells mock-infected or infected with the PR8 virus. The lactylome analysis revealed that 1,295 host proteins (2,792 sites) were significantly upregulated, and 69 host proteins (105 sites) were significantly downregulated upon the IAV infection (Fig. S5A). Notably, 37 sites on eight viral proteins (NP, PA, PB1, PB2, HA, M1, M2, and NS1) were identified to become lactylated during IAV infection (Fig. S5B). These results indicate that viral NP, PA, PB1, and PB2 proteins undergo lactylation during IAV infection.

### SIRT1 dampens the lactylation of IAV NP, PA, and PB2 proteins

**Fig 6:**
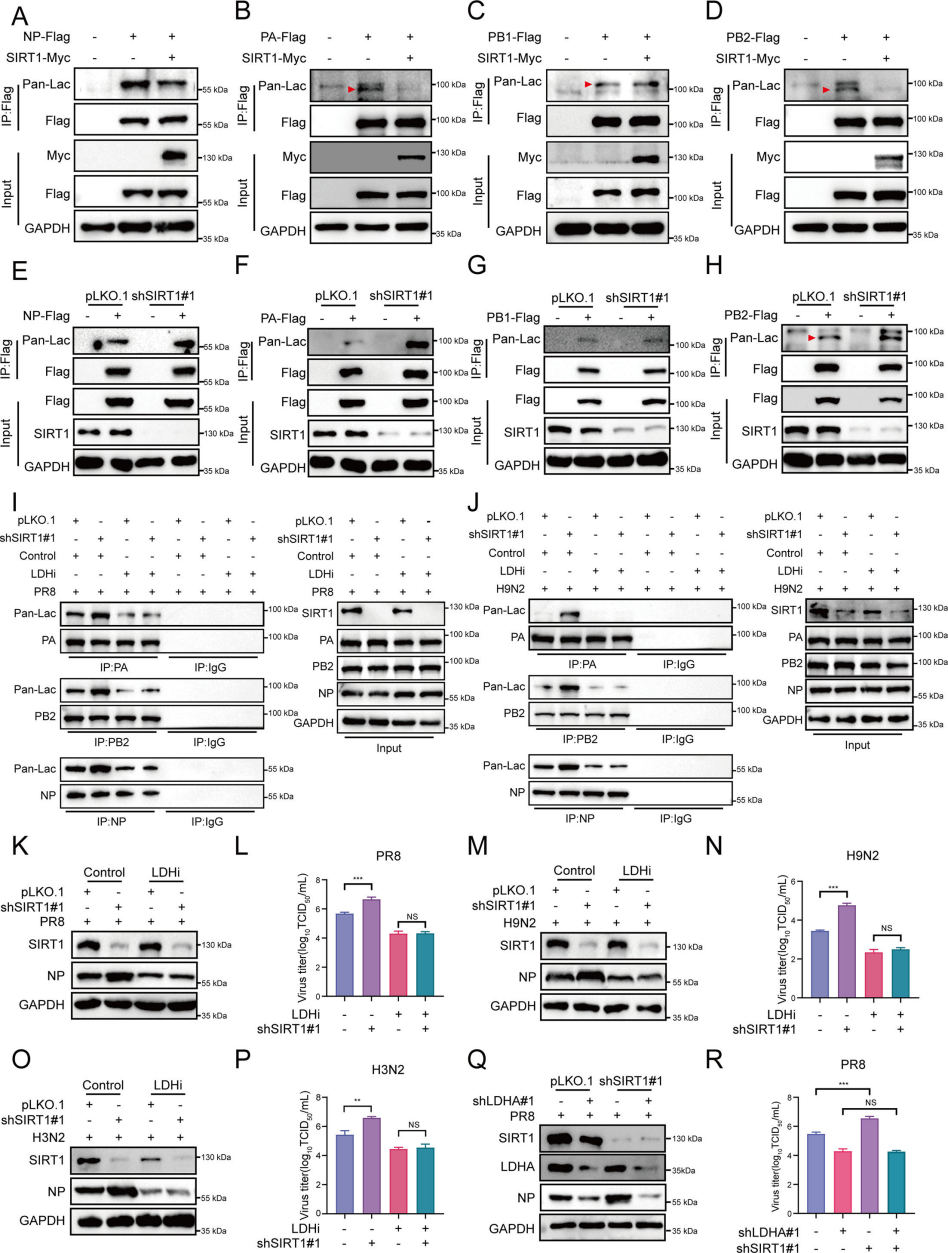
SIRT1 suppresses IAV replication via delactylation of viral NP, PA, and PB2 proteins. (**A–D**) Flag-tagged NP (**A**), PA (**B**), PB1 (**C**) or PB2 (**D**), along with SIRT1-Myc or EV, were transfected into HEK293T cells. IP assays were performed using anti-Flag antibody, followed by Western blot analysis to detect lactylation of these viral proteins using anti-pan lac antibody. (**E–H**) Control and SIRT1 knockdown cells were transfected with Flag-tagged NP (**E**), PA (**F**), PB1 (**G**) or PB2 (**H**), and IP assays were performed using anti-Flag antibody, followed by Western blot to detect lactylation of indicated viral proteins. (**I and J**) Control and SIRT1 knockdown A549 cells were pretreated with oxamate (LDHi) (50 mM) for 1 h. To ensure equal amounts of viruses are present in different samples, control A549 cells were infected with PR8 (MOI = 0.1) (**I**) or H9N2 (MOI = 0.2) (**J**). SIRT1 knockdown A549 cells were infected with PR8 (MOI = 0.03) or H9N2 (MOI = 0.05). Control A549 cells with oxamate (LDHi) treatment were infected with PR8 (MOI = 0.5) or H9N2 (MOI = 0.6), while SIRT1 knockdown A549 cells with oxamate (LDHi) treatment were infected with PR8 (MOI = 0.1) or H9N2 (MOI = 0.1) for 24 h. IP assays were performed using anti-NP, anti-PA, or anti-PB2 antibody, with IgG as a negative control, followed by Western blot analysis using anti-pan lac antibody. (**K–P**) Control and SIRT1 knockdown A549 cells were pretreated with oxamate (LDHi) (50 mM) for 1 h, followed by infection with A/PR8/34 (H1N1) (MOI = 0.01) (**K and L**), H9N2 (MOI = 0.01) (**M and N**), or H3N2 subtype virus (MOI = 0.01) (**O and P**) for 24 h. Viral NP protein levels were examined by Western blot (**K, M, and O**). Viral titers in the supernatants were determined by TCID_50_ assays (**L, N, and P**). (**Q and R**) Control and SIRT1 knockdown A549 cells were infected with lentiviruses expressing control or LDHA shRNA, followed by PR8 virus infection. Viral NP protein levels were detected by Western blot (**Q**). Viral titers in the supernatants were determined by TCID_50_ assays (**R**). Data are presented as mean ± SD of three independent experiments. **P < 0.01 and ***P < 0.001.

SIRT1 has been recently reported as a robust lysine delactylase (54), and our above results suggest that SIRT1 interacts with viral NP, PA, PB1, and PB2 proteins that undergo lactylation following IAV infection, thus we asked whether SIRT1 might regulate IAV replication by modulating lactylation of these viral proteins. To test this, we first evaluated the effect of SIRT1 overexpression on the lactylation of viral NP, PA, PB1, and PB2 proteins. Control and SIRT1 overexpressing cells were transfected with Flag-tagged NP, PA, PB1, or PB2 respectively, followed by IP assays and Western blot to detect lactylation of these viral proteins. An obvious decrease in the lactylation levels of NP, PA, and PB2 was observed in SIRT1 overexpressing cells compared with those in control cells (Fig. 6A, B, and D). In contrast, comparable lactylation levels of PB1 were detected between SIRT1 overexpressing and control cells (Fig. 6C). These data indicate that overexpression of SIRT1 dampens lactylation of IAV NP, PA, and PB2 proteins. On the other hand, we also examined the effect of SIRT1 knockdown on the lactylation of NP, PA, PB1, and PB2 proteins. Control and SIRT1 knockdown cells were transfected with Flag-tagged NP, PA, PB1, or PB2, respectively, followed by IP assays and Western blot to detect lactylation of these viral proteins. As shown in Fig. 6E through H, depletion of SIRT1 led to a significant increase in the lactylation levels of NP, PA, and PB2, but not PB1, compared with the control cells.

Next, we further addressed the regulation of NP, PA, and PB2 lactylation by SIRT1 during actual IAV infection. Control and SIRT1 overexpressing A549 cells were infected with influenza virus A/PR8/34 (H1N1), and IP assays were performed using anti-NP, PA, or PB2 antibody with IgG as a negative control, followed by Western blot to detect the lactylation of NP, PA, or PB2 in the cell lysates. As shown in Fig. S6A, significantly decreased lactylation levels of NP, PA, and PB2 were observed in SIRT1 overexpressing cells compared with those in control cells following PR8 virus infection. On the other hand, control and SIRT1 knockdown A549 cells were infected with influenza virus A/PR8/34 (H1N1), H3N2, or H9N2, respectively, and the lactylation of NP, PA, or PB2 was examined. Our data showed that depletion of SIRT1 led to a significant increase in the lactylation levels of NP, PA, and PB2 compared with control cells following infection with PR8 (H1N1), H3N2, and H9N2 IAVs (Fig. 6I and J; Fig. S6J). These results suggest that SIRT1 mediates delactylation of viral NP, PA, and PB2 proteins, thereby inhibiting the vRNP activity and the IAV replication.

### SIRT1 suppresses IAV replication via delactylation of viral NP, PA, and PB2 proteins

Next, we asked whether delactylation of viral proteins was involved in the regulation of IAV replication by SIRT1. LDHA is a major enzyme mediating the production of lactate, which may in turn promote protein lactylation (25). We employed an LDH inhibitor (LDHi) that could decrease lactate levels and consequent protein lactylation (25). Control and SIRT1 knockdown cells were treated with the LDH inhibitor and then infected with influenza viruses. The viral replication was determined by Western blot and TCID_50_ assays. As shown in Fig. S6B through I, treatment with the LDH inhibitor significantly decreased the whole protein lactylation and reduced the IAV replication in the cells.

Next, we further evaluated the effect of the LDH inhibitor on the upregulation of IAV replication caused by SIRT1 depletion. When the control and SIRT1 knockdown cells were untreated with the LDH inhibitor, depletion of SIRT1 led to a significant increase in the lactylation of NP, PA, and PB2 compared with the control following infection with IAV PR8 (Fig. 6I), H9N2 (Fig. 6J), or H3N2 (Fig. S6J). Accordingly, enhanced NP, PA, and PB2 protein levels and increased virus titers were observed in the SIRT1 knockdown cells compared with those in control cells following IAV infections (Fig. 6K through P; Fig. S6K through M). These data indicate that depletion of SIRT1 promotes IAV replication through increasing the lactylation of viral NP, PA, and PB2 proteins. In contrast, when the control and SIRT1 knockdown cells were treated with the LDH inhibitor, we found that treatment with the LDH inhibitor led to a significant decrease in the NP, PA, and PB2 lactylation, and comparable lactylation levels of NP, PA, and PB2 were observed between control and SIRT1 knockdown cells infected with IAV PR8 (Fig. 6I), H9N2 (Fig. 6J), or H3N2 (Fig. S6J). In line with this, knockdown of SIRT1 failed to enhance the replication of the viruses in the cells, indicating that the upregulation of IAV replication caused by SIRT1 depletion was abolished by the LDH inhibitor treatment (Fig. 6K through P; Fig. S6K through M). Additionally, we generated LDHA knockdown A549 cells and evaluated the effect of LDHA deficiency on the regulation of IAV replication by SIRT1. As shown in Fig. 6Q through R and Fig. S6N, the enhancement of IAV replication caused by SIRT1 knockdown was significantly attenuated by depletion of LDHA in the cells. Moreover, we observed that inhibition or depletion of LDHA decreased the expression of SIRT1, and MG132, a selective inhibitor of proteasome, but not the lysosome inhibitor chloroquine (CQ), could restore the reduced SIRT1 protein levels in the LDH inhibitor-treated cells (Fig. S6O and P), implying that the ubiquitin–proteasome pathway may be involved in the decreased SIRT1 protein levels caused by inhibition and depletion of LDHA. Together, these results suggest that SIRT1 suppresses IAV replication via delactylation of the viral NP, PA, and PB2 proteins.

## DISCUSSION

SIRT1, a member of sirtuins (SIRTs) that belongs to the class III histone deacetylase family, has been reported to play important roles in modulating immune and inflammatory responses following bacterial, viral, and parasitic infections (59, 70, 71). For instance, SIRT1 restrains type I IFN and type II IFN signaling and the antiviral immunity by deacetylating STAT1 and STAT3, thereby downregulating the phosphorylation of STAT1 (Y701) and STAT3 (Y705) (72). In addition, SIRT1 interacts with IFI16 and decreases the acetylation of IFI16, leading to the inhibition of IFI16 cytoplasmic localization and impairment of antiviral responses against DNA virus (73). Moreover, SIRT1 can regulate interferon production by modulating IRF3/IRF7 liquid–liquid phase separation (LLPS) (58). In the present study, we revealed SIRT1 as a negative regulator for IAV replication. Depletion of SIRT1 significantly promoted the replication of IAV, whereas overexpression of SIRT1 inhibited the viral replication. Importantly, we found that IAV infection led to a significant decrease in the expression of SIRT1. These findings suggest that IAV has evolved a mechanism to downregulate the expression of SIRT1 to benefit its replication in the host cells. In an attempt to probe the molecular mechanisms by which IAV downregulates SIRT1 expression, we found that overexpression of PA, but not other viral proteins, led to a decrease in the SIRT1 protein levels, implying that PA might be involved in the downregulation of SIRT1 by IAV infection. Further studies are deserved to elucidate the precise mechanisms underlying the regulation of SIRT1 by PA during IAV infection.

A distinguishing feature of the IAV life cycle is that transcription and replication of the viral genome occur in the nucleus of infected cells, which is orchestrated by the vRNP complex (9, 11, 74, 75). During the early phase of IAV infection, the vRNP complex is released into the cytoplasm and translocated to the nucleus, following completion of endocytosis and uncoating (76, 77). Although progress has been made to address molecular mechanisms accounting for the migration of the vRNP complex during IAV infection, detailed mechanisms underlying the regulation of the vRNP activity are not fully understood. In an attempt to decode the mechanisms underlying SIRT1-mediated suppression of IAV replication, we examined the interaction between SIRT1 and IAV proteins and found that SIRT1 interacted with NP, PA, PB1, and PB2, components of the vRNP complex. We further evaluated the effect of SIRT1 on the vRNP activity. Our data showed that forced expression of SIRT1 significantly reduced the vRNP activity, thereby inhibiting the transcription and replication of the IAV genome, which can explain the attenuated replication of IAV in SIRT1 overexpressing cells.

Aerobic glycolysis is a key feature of many viral infections, leading to substantial accumulation of lactate (34). Therefore, the role of lactylation in viral pathogenesis and host antiviral responses has attracted increasing attention (28, 69, 78, 79). However, the relationship between lactylation and IAV infection, as well as the functional involvement of this relationship in the complicated interplay between the host and IAV, remains poorly understood. In addition, previous studies have mainly focused on the lactylation of host factors. Lactylation of viral proteins and their potential roles in the viral pathogenesis remain largely unexplored. Recently, lactylation of the SARS-CoV-2 spike (S) protein has been revealed to promote the association of S protein with the host receptor ACE2 or the host protease TMPRSS2, thereby facilitating the viral entry and infection (80). Another study shows that HCMV infection induces lactylation of the viral proteins, and lactylation of HCMV protein can modulate the processes involved in the viral replication. These observations indicate that lactylation of viral proteins plays important roles in the viral infection and pathogenesis. Here, we identified that viral NP, PA, PB1, and PB2 proteins underwent lactylation during IAV infection. Of note, we found that SIRT1 promoted the delactylation of NP, PA, and PB2 proteins, as depletion of SIRT1 enhanced the lactylation of NP, PA, and PB2, while overexpression of SIRT1 obviously reduced lactylation of these viral proteins. These results suggest a mechanism that SIRT1 interacts with and mediates the delactylation of viral NP, PA, and PB2 proteins, thereby interfering with the vRNP activity and ultimately mitigating the IAV replication. Interestingly, the expression of SIRT1 was significantly decreased following IAV infection, implying that IAV had evolved a mechanism to downregulate the SIRT1 expression, thereby ensuring appropriate lactylation of viral proteins and facilitating the IAV replication. Additionally, we performed a lactylome analysis and identified 37 potential lactylation sites on eight viral proteins during IAV infection. The precise mechanisms by which lactylation modulates the function of these viral proteins and the IAV pathogenesis deserve further investigation.

Notably, we observed that overexpression and depletion of SIRT1 had no significant effect on the lactylation of viral PB1 protein that was overexpressed in the HEK293T cells. Lactylation is a reversible biological process and dynamically regulated by the writers that catalyze protein lactylation and erasers that remove protein lactylation. Therefore, the lactylation and delactylation of viral proteins are highly dependent on the expression and activity of lactylation writers and erasers during IAV infection, implying that the lactylation status of viral proteins may vary at different stages of the viral infection. Moreover, lactylation and other post-translational modifications (PTMs) may occur at the same protein or even at the same sites and serve distinct functions or exhibit precise interplay under specific conditions. For instance, lactylation and acetylation on the same protein sites might have distinct functions or exhibit competition (81, 82). Therefore, other PTMs may influence the lactylation and delactylation of viral proteins during IAV infection. Further studies are warranted to decipher the detailed mechanisms underlying lactylation/delactylation of viral proteins during IAV infection, and the crosstalk between lactylation and other PTMs of viral proteins.

Collectively, our studies reveal that viral NP, PA, PB1, and PB2 proteins undergo lactylation following IAV infection. Moreover, SIRT1 interacts with and promotes the delactylation of NP, PA, and PB2, thereby restraining the vRNP activity and attenuating the IAV replication. These findings establish a critical role for SIRT1-mediated delactylation of viral proteins in regulating IAV infection and provide an important insight into the complicated interplay between the host and IAV.

## Data Availability

The data and materials are available from corresponding author on reasonable request. The lactylome data are available in Zenodo under doi 10.5281/zenodo.18247436.

## SUPPLEMENTAL MATERIAL

Supplemental material mbio.02489-25-s0001.pdf Supplemental figures and table.

ASM does not own the copyrights to Supplemental Material that may be linked to, or accessed through, an article. The authors have granted ASM a non-exclusive, world-wide license to publish the Supplemental Material files. Please contact the corresponding author directly for reuse.
