# Supplementary material for: Delactylation of viral proteins by SIRT1 suppresses influenza A virus replication
Source: mBio. 2026 Mar 18;17(4):e02489-25. doi: 10.1128/mbio.02489-25 (PMC13059713; doi:10.1128/mbio.02489-25)
Supplement: Supplemental material — Supplemental figures and table. [file mbio.02489-25-s0001.pdf]

Supplemental Figure

Delactylation of viral proteins by SIRT1 suppresses influenza A virus replication

Dongdong Chen[1,2,#], Gejie Zhao[1,2,#], Jia Zhou[1,2,#], Peng Sun[1,#], Shutong He[1], Changjie Lv[1,2], Yuhai Chen[3], Shouhai Zhu[4], Ming Gao[5,*], and Guijie Guo[1,2,6,*]

[1]Key Laboratory of Animal Pathogen Infection and Immunology of Fujian Province, College of Animal Sciences, Fujian Agriculture and Forestry University, Fuzhou 350002, China.

[2]Fujian Province Joint Laboratory of Animal Pathogen Prevention and Control of the “Belt and Road”, College of Animal Sciences, Fujian Agriculture and Forestry University, Fuzhou 350002, China.

[3]CAS Key Laboratory of Pathogenic Microbiology and Immunology, Institute of Microbiology, Chinese Academy of Sciences (CAS), Beijing 100101, China.

[4]Department of Oncology, Mayo Clinic, Rochester, MN 55905, USA.

[5]State Key Laboratory of Environmental Chemistry and Ecotoxicology, Research Center for Eco-Environmental Sciences, Chinese Academy of Sciences (CAS), Beijing 100101, China.

[6]Engineering Research Center for Animal Breeding and Sustainable Production, College of Animal Sciences, Fujian Agriculture and Forestry University, Fuzhou 350002, China.

#These authors contributed equally to this work.

*Correspondence:

Ming Gao, minggao@rcees.ac.cn;

Guijie Guo, guoguijie@fafu.edu.cn, or guojie1125@163.com

Figure S1

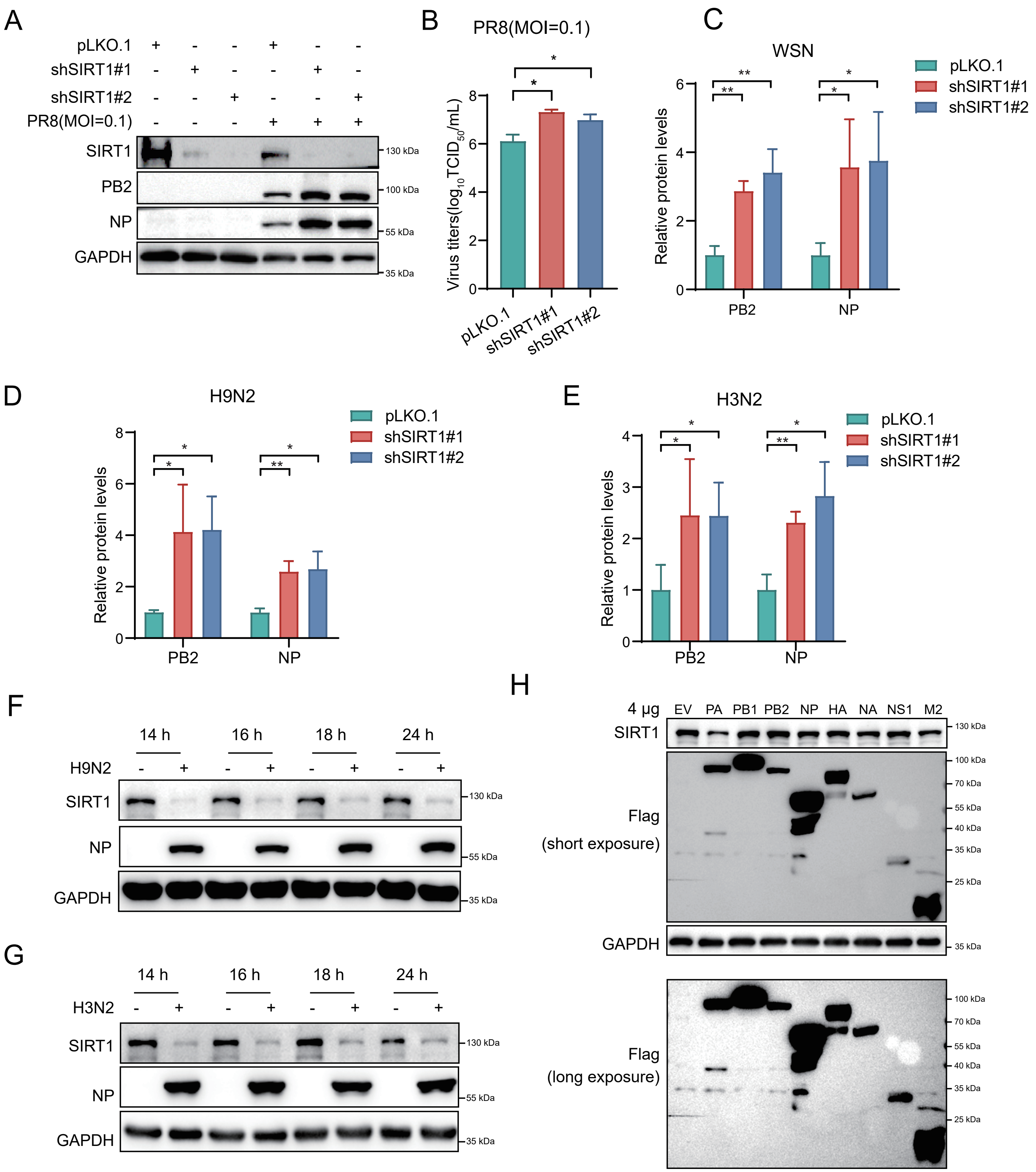

A
pLKO.1
shSIRT1#1
shSIRT1#2
PR8(MOI=0.1)
SIRT1
PB2
NP
GAPDH
130 kDa
100 kDa
55 kDa
35 kDa
B
PR8(MOI=0.1)
Virus titers(log10TCID50/mL)
pLKO.1
shSIRT1#1
shSIRT1#2
C
WSN
Relative protein levels
PB2
NP
D
H9N2
E
H3N2
F
14 h
16 h
18 h
24 h
H9N2
G
H3N2
H
4 μg
EV
PA
PB1
PB2
NP
HA
NA
NS1
M2
Flag
(short exposure)
(long exposure)
70 kDa
40 kDa
25 kDa

Figure S1 SIRT1 inhibits the replication of IAV

(A-B) Control and SIRT1 knockdown A549 cells were infected with influenza virus A/PR8/34 (H1N1) (MOI = 0.1) for 24 h. Viral NP and PB2 protein levels were examined by Western blot (A). Viral titers in the supernatants were determined by $TCID_{50}$ assays (B). **(C-E)** Control and SIRT1 knockdown A549 cells were infected with influenza virus A/WSN/33 (H1N1) (MOI = 0.01) (C), H9N2 (MOI = 0.01) (D), or H3N2 virus (MOI = 0.01) (E) for 24 h. The band intensities of NP and PB2 proteins were analyzed by image J software. **(F-G)** A549 cells were infected with IAV H9N2 (MOI = 0.01) (F), or H3N2 virus (MOI = 0.01) (G) for 14, 16, 18 and 24 h. SIRT1 and viral NP protein levels were examined by Western blot. **(H)** HEK293T cells were transfected with Flag tagged-NA, HA, PA, NS1, NP, PB1, PB2, M2, or EV (4 μg) for 24 h. SIRT1 protein levels were examined by Western blot. Data are presented as mean ± SD of three independent experiments. *$p < 0.05$, **$p < 0.01$.

Figure S2

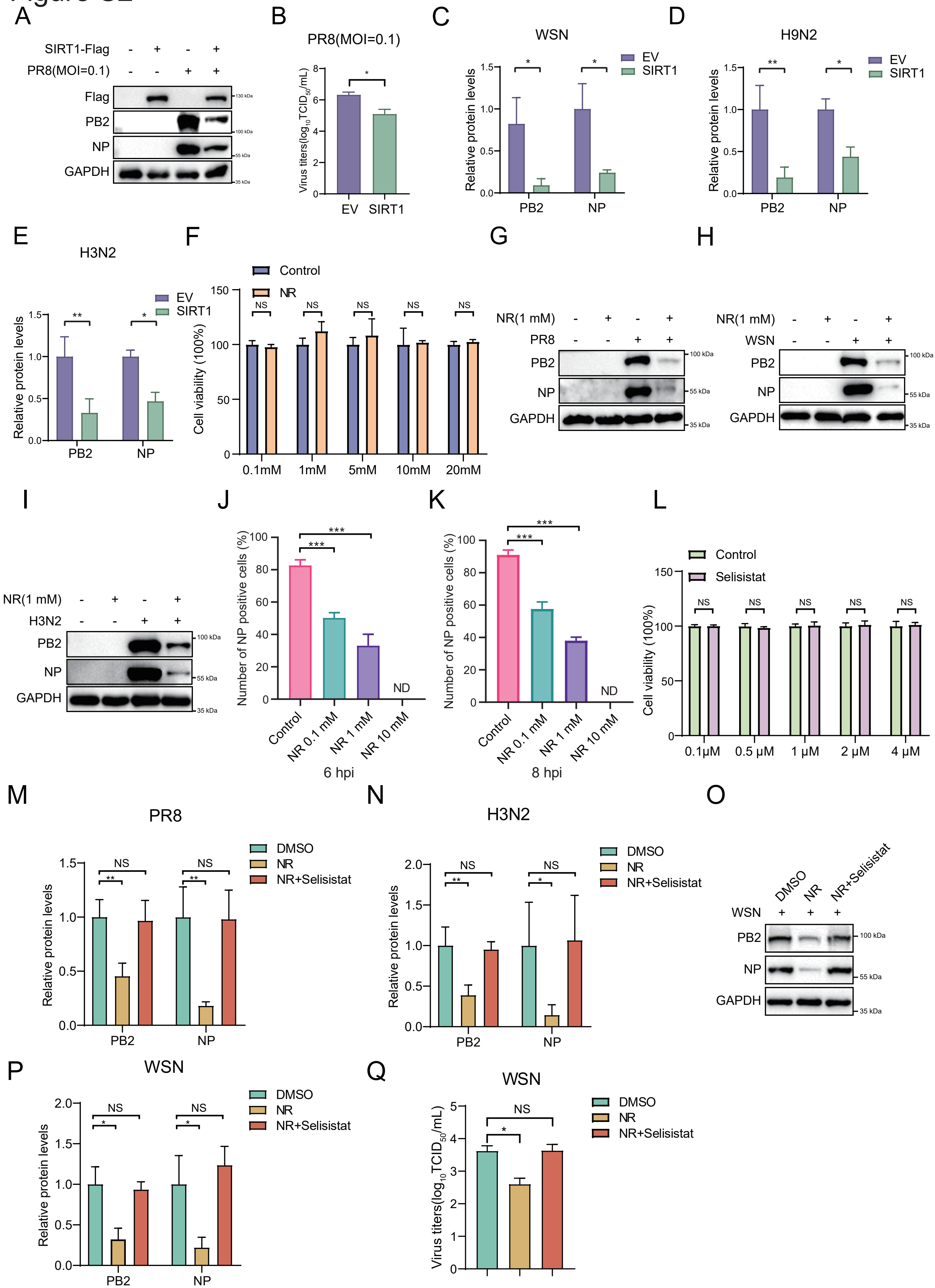

A
SIRT1-Flag
PR8(MOI=0.1)
Flag
PB2
NP
GAPDH
B
PR8(MOI=0.1)
Virus titers(log10TCID50/mL)
EV
SIRT1
C
WSN
D
H9N2
E
H3N2
Relative protein levels
EV
SIRT1
PB2
NP
F
Control
NR
Cell viability (100%)
0.1mM
1mM
5mM
10mM
20mM
G
NR(1 mM)
PR8
H
WSN
I
H3N2
J
K
Number of NP positive cells (%)
Control
NR 0.1 mM
NR 1 mM
NR 10 mM
ND
6 hpi
8 hpi
L
Selisistat
0.1μM
0.5 μM
1 μM
2 μM
4 μM
M
PR8
N
O
P
Q
DMSO
NR+Selisistat

Figure S2 Activation of SIRT1 represses IAV replication

(A-E) Control and SIRT1 overexpressing A549 cells were infected with influenza virus A/PR8/34 (H1N1) (MOI = 0.1) (A-B), A/WSN/33 (H1N1) (MOI = 0.01) (C), H9N2 subtype virus (MOI = 0.01) (D), or H3N2 subtype virus (MOI = 0.01) (E) for 24 h. Viral NP and PB2 protein levels were detected by Western blot (A). Viral titers in the supernatants were determined by $TCID_{50}$ assays (B). The band intensities of NP and PB2 proteins were analyzed by image J software (C-E). **(F)** The cytotoxicity of NR (0.1, 1, 5, 10, and 20 mM) in A549 cells after 24 h treatment, was tested using CCK8 assays. **(G-I)** A549 cells were pretreated with NR (1 mM) for 1 h, followed by infection with PR8 (G), WSN (H), or H3N2 (I) virus (MOI = 0.01). Viral NP and PB2 protein levels were measured by Western blot at 24 hpi. **(J-K)** A549 cells were pretreated with NR (0.1 mM, 1 mM or 10 mM) for 1 h, followed by infection with PR8 virus (MOI = 5). The cells were then fixed and stained with anti-NP antibody, followed by incubation with the secondary antibody. The nuclei were stained with DAPI (Figure 2L). NP positive cells in the control and NR-treated cells were quantified at 6 hpi (J) and 8 hpi (K). **(L)** A549 cells were incubated with control or Selisistat (0.1, 0.5, 1, 2, and 4 μM) for 24 h, followed by CCK8 assays. **(M-Q)** A549 cells were pretreated with NR (1 mM) and Selisistat (1 μM) for 1 h, followed by infection with PR8 (M), H3N2 (N), or WSN virus (O-Q) (MOI = 0.01). Viral NP and PB2 protein levels were detected by Western blot at 24 hpi (O). Viral titers in the supernatants were determined by $TCID_{50}$ assays (Q). The band intensities of NP and PB2 proteins were analyzed by image J software (M, N, P). Data are presented as mean ± SD of three independent experiments. $^{*}p < 0.05$, $^{**}p < 0.01$, $^{***}p < 0.001$.

Figure S3

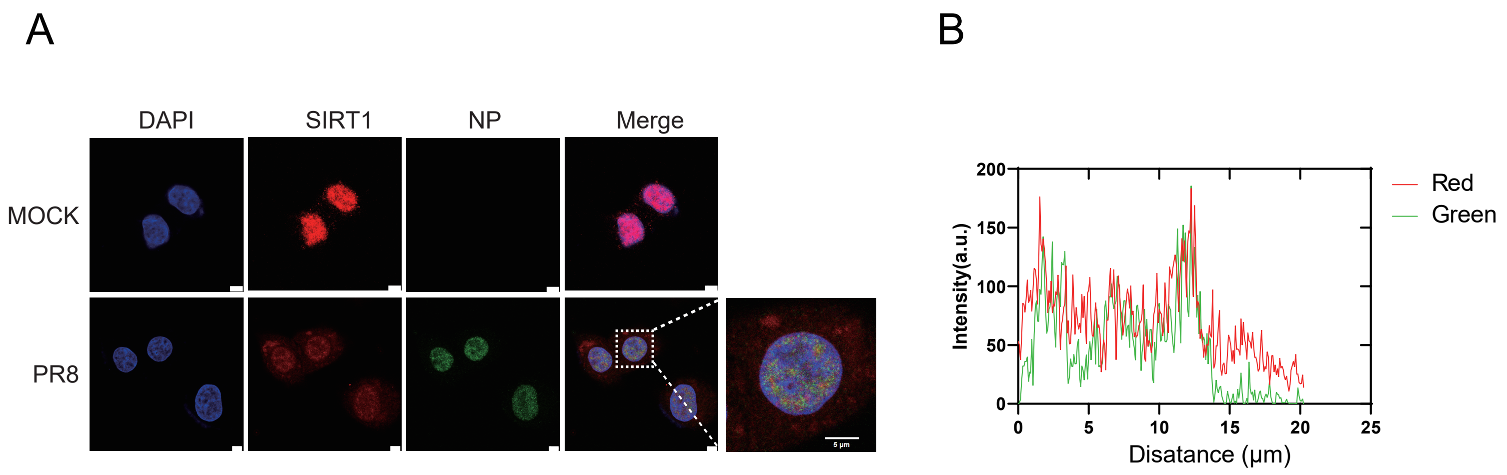
A
DAPI
SIRT1
NP
Merge
MOCK
PR8
B
Red
Green
Intensity(a.u.)
Disatance (μm)
200
150
100
50
0
0
5
10
15
20
25

Figure S3 SIRT1 colocalizes with NP during IAV infection

(A-B) A549 cells were infected with PR8 virus (MOI = 0.01) for 16 h, and then subjected to immunofluorescence assays using an anti-SIRT1 antibody (red) and an anti-NP antibody (green). DAPI was used to stain the nucleus (blue) (A). The co-localization of SIRT1 and NP protein were analyzed by image J software (B).

Figure S4

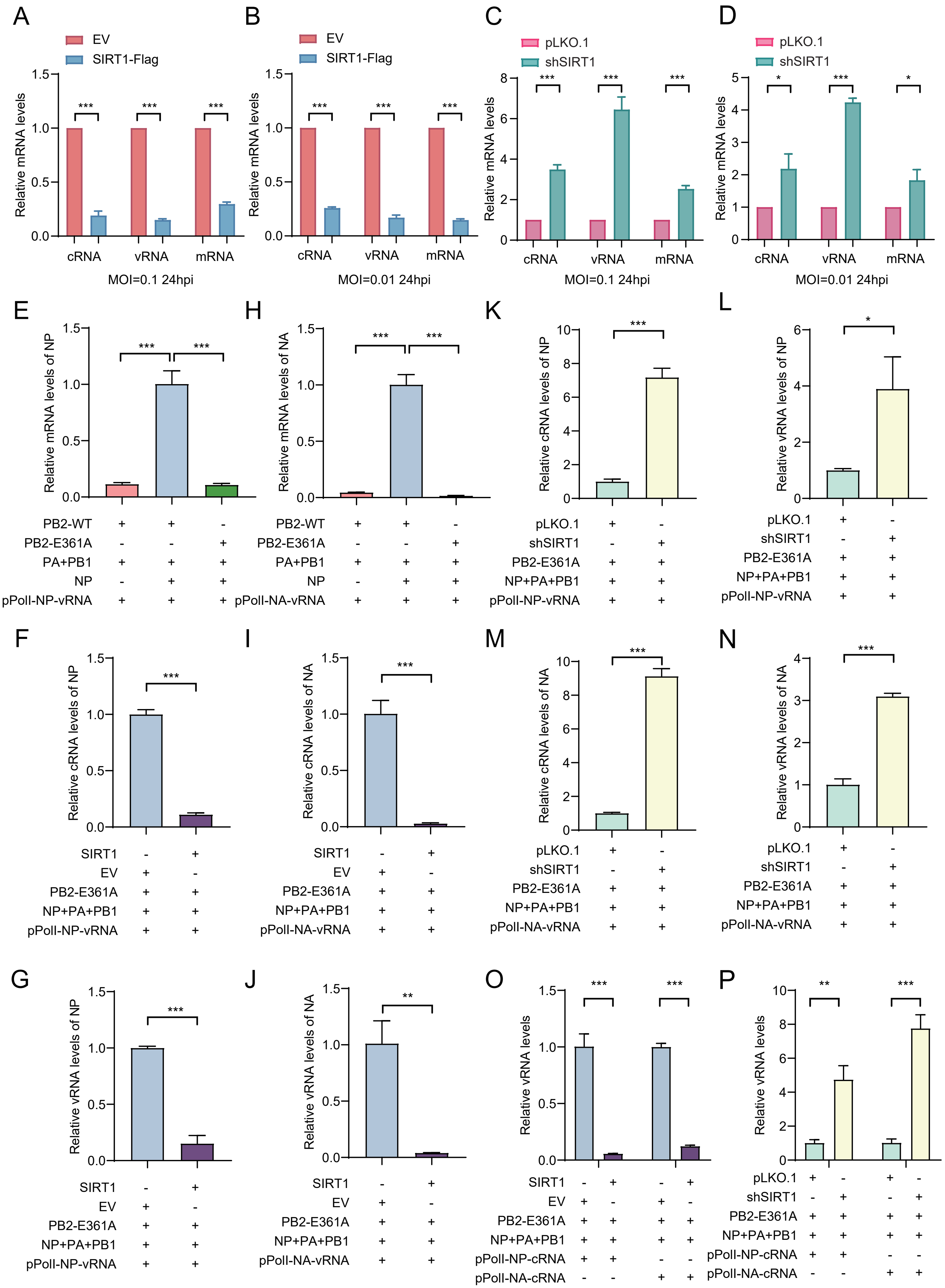

A
EV
SIRT1-Flag
Relative mRNA levels
cRNA
vRNA
mRNA
MOI=0.1 24hpi
B
EV
SIRT1-Flag
Relative mRNA levels
cRNA
vRNA
mRNA
MOI=0.01 24hpi
C
pLKO.1
shSIRT1
Relative mRNA levels
cRNA
vRNA
mRNA
MOI=0.1 24hpi
D
pLKO.1
shSIRT1
Relative mRNA levels
cRNA
vRNA
mRNA
MOI=0.01 24hpi
E
Relative mRNA levels of NP
PB2-WT
PB2-E361A
PA+PB1
NP
pPolI-NP-vRNA
H
Relative mRNA levels of NA
PB2-WT
PB2-E361A
PA+PB1
NP
pPolI-NA-vRNA
K
Relative cRNA levels of NP
pLKO.1
shSIRT1
PB2-E361A
NP+PA+PB1
pPolI-NP-vRNA
L
Relative vRNA levels of NP
pLKO.1
shSIRT1
PB2-E361A
NP+PA+PB1
pPolI-NP-vRNA
F
Relative cRNA levels of NP
SIRT1
EV
PB2-E361A
NP+PA+PB1
pPolI-NP-vRNA
I
Relative cRNA levels of NA
SIRT1
EV
PB2-E361A
NP+PA+PB1
pPolI-NA-vRNA
M
Relative cRNA levels of NA
pLKO.1
shSIRT1
PB2-E361A
NP+PA+PB1
pPolI-NA-vRNA
N
Relative vRNA levels of NA
pLKO.1
shSIRT1
PB2-E361A
NP+PA+PB1
pPolI-NA-vRNA
G
Relative vRNA levels of NP
SIRT1
EV
PB2-E361A
NP+PA+PB1
pPolI-NP-vRNA
J
Relative vRNA levels of NA
SIRT1
EV
PB2-E361A
NP+PA+PB1
pPolI-NA-vRNA
O
Relative vRNA levels
SIRT1
EV
PB2-E361A
NP+PA+PB1
pPolI-NP-cRNA
pPolI-NA-cRNA
P
Relative vRNA levels
pLKO.1
shSIRT1
PB2-E361A
NP+PA+PB1
pPolI-NP-cRNA
pPolI-NA-cRNA

Figure S4 SIRT1 inhibits viral transcription and replication

(A-B) Control and SIRT1 overexpressing A549 cells were infected with PR8 virus at an MOI of 0.1 (A) or 0.01 (B). Total RNA was collected at 24 hpi. NP vRNA, cRNA, and mRNA levels were analyzed by RT-qPCR. **(C-D)** Control and SIRT1 knockdown A549 cells were infected with PR8 virus at an MOI of 0.1 (C) or 0.01 (D). Total RNA was collected at 24 hpi. NP vRNA, cRNA, and mRNA levels were analyzed by RT-qPCR. **(E-J)** HEK293T cells were transfected with either pPol I-NA-vRNA or pPol I-NP-vRNA, along with plasmids encoding PB2 (E361A), PB1, PA, NP, and Flag-SIRT1 or EV. Total RNA was collected at 24 h. NP (E-G) or NA (H-J) vRNA, cRNA, and mRNA levels were analyzed by RT-qPCR. **(K-N)** Control and SIRT1 knockdown HEK293T cells were transfected with either pPol I-NA-vRNA or pPol I-NP-vRNA, along with plasmids encoding PB2 (E361A), PB1, PA, and NP. Total RNA was collected after 24 h. NP (K, L) or NA (M, N) vRNA, cRNA, and mRNA levels were analyzed by RT-qPCR. **(O)** Control and SIRT1 overexpressing HEK293T cells were transfected with either pPol I-NA-cRNA or pPol I-NP-cRNA, along with plasmids encoding PB2 (E361A), PB1, PA, and NP. The levels of NP or NA vRNA were measured at 24 h after the transfection. **(P)** Control and SIRT1 knockdown HEK293T cells were transfected with either pPol I-NP-cRNA or pPol I-NA-cRNA, along with plasmids encoding PB2 (E361A), PB1, PA, and NP. The levels of NP or NA vRNA were detected at 24 h after the transfection. Data are shown as means ± SD (n = 3). $*p < 0.05$, $**p < 0.01$, $***p < 0.001$.

Figure S5

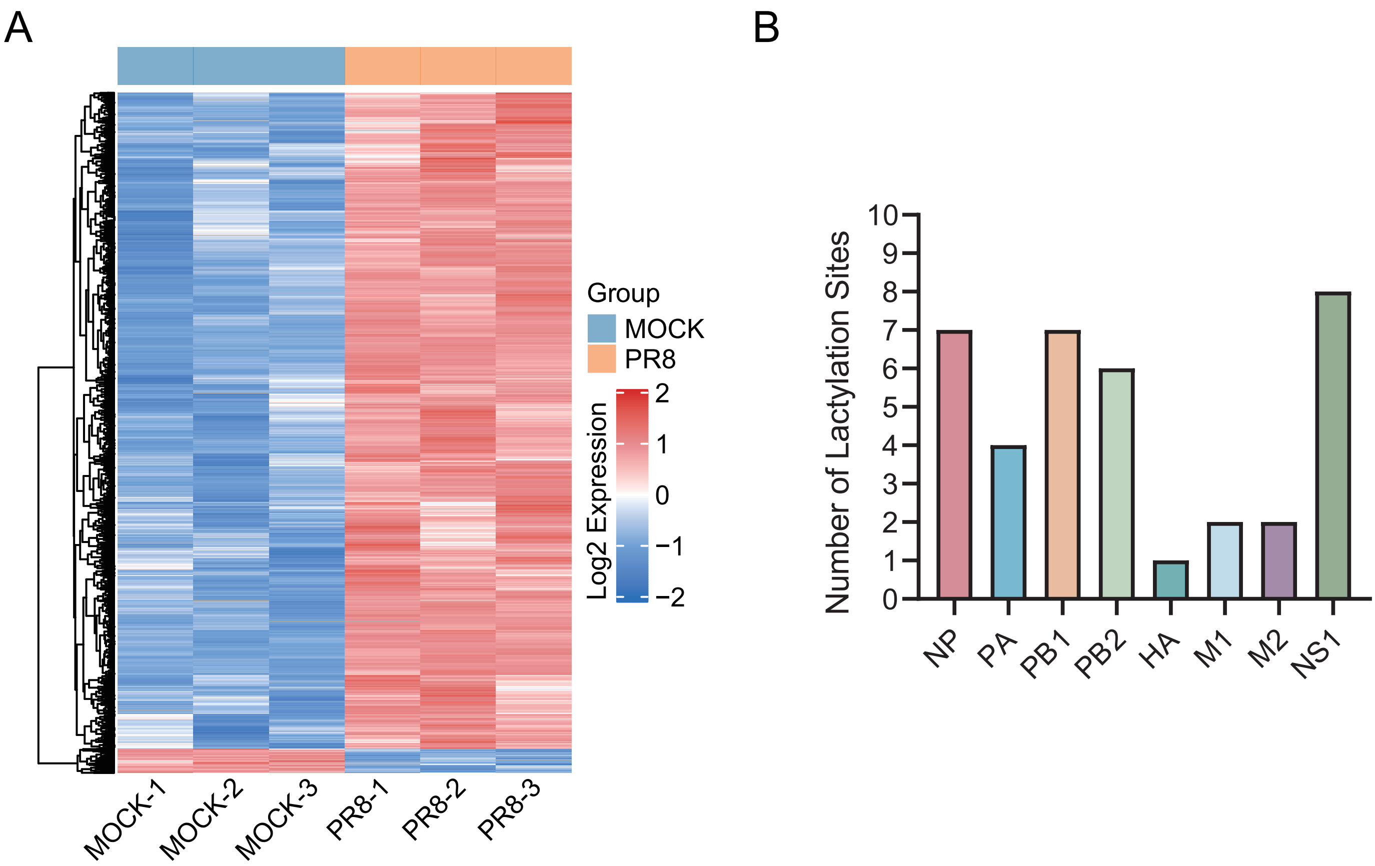
A
Group
MOCK
PR8
Log2 Expression
2
1
0
−1
−2
MOCK-1
MOCK-2
MOCK-3
PR8-1
PR8-2
PR8-3
B
Number of Lactylation Sites
10
9
8
7
6
5
4
3
2
1
0
NP
PA
PB1
PB2
HA
M1
M2
NS1

Figure S5 Viral proteins become lactylated during IAV infection

(A) Lactylome analysis was conducted on A549 cells infected with or without influenza virus A/PR8/34 (H1N1). Shown are differentially lactylation sites of host proteins in A549 cells following PR8 virus infection. **(B)** The number of lactylation sites in viral proteins.

Figure S6

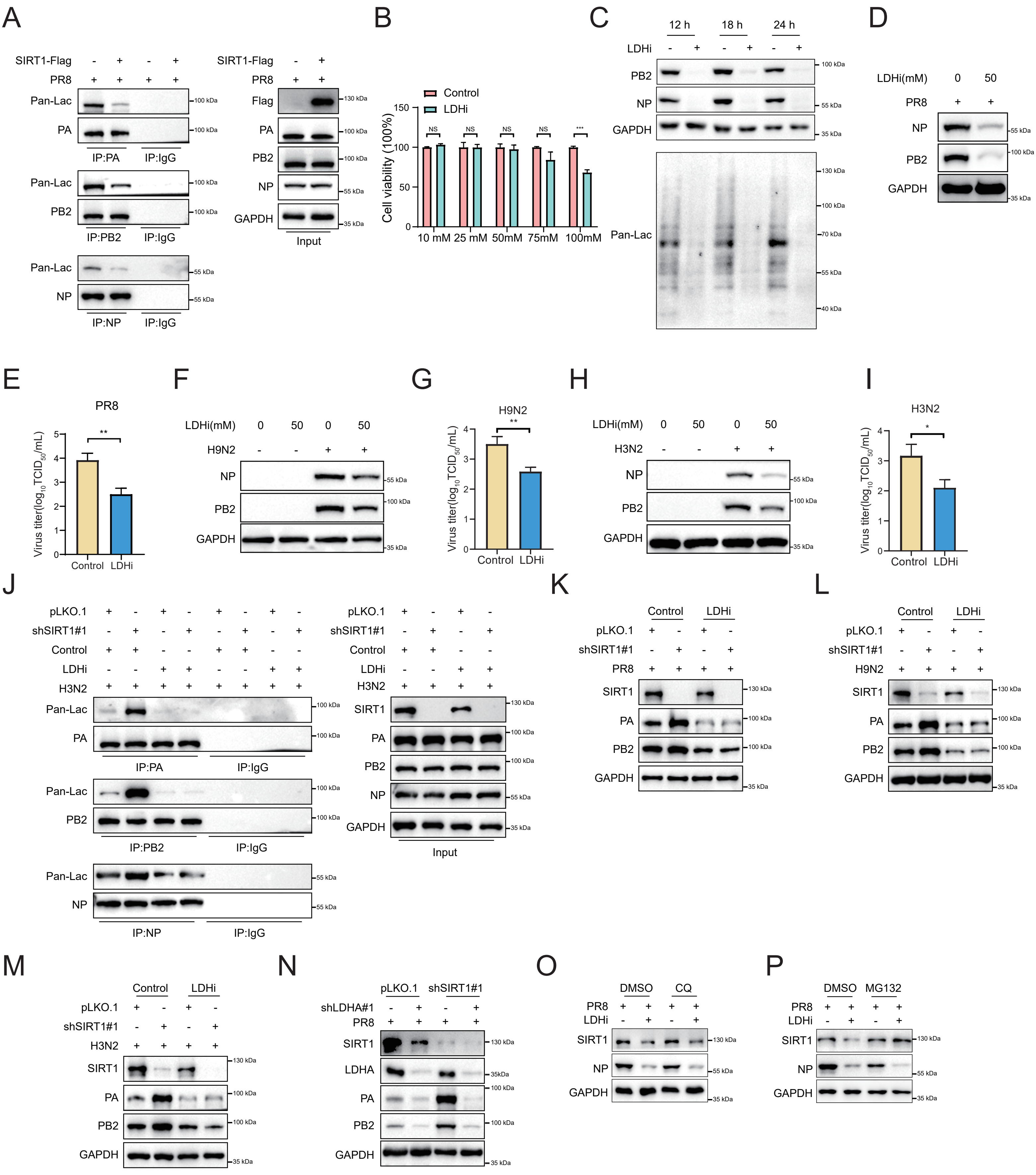

A
SIRT1-Flag
PR8
Pan-Lac
PA
IP:PA
IP:IgG
PB2
IP:PB2
NP
IP:NP
Flag
GAPDH
Input
B
Control
LDHi
Cell viability (100%)
NS
10 mM
25 mM
50mM
75mM
100mM
C
12 h
18 h
24 h
D
LDHi(mM)
E
Virus titer($log_{10}TCID_{50}$/mL)
F
H9N2
G
H
H3N2
I
J
pLKO.1
shSIRT1#1
SIRT1
K
L
M
N
shLDHA#1
LDHA
O
DMSO
CQ
P
MG132

Figure S6 SIRT1 suppresses IAV replication via delactylation of viral NP, PA, and PB2 proteins

(A) Control and SIRT1 overexpressing A549 were infected with PR8 virus at an MOI = 0.1 or MOI = 1 respectively. IP assays were performed using anti-NP, anti-PA, or anti-PB2 antibody, with IgG as a negative control, followed by Western blot using anti-pan lac antibody. **(B)** The cytotoxicity of oxamate (LDHi) (10, 25, 50, 75, and 100 mM) in A549 cells after 24 h treatment was tested using CCK8 assays. **(C)** A549 cells were pretreated with oxamate (LDHi) (50 mM) for 1 h, followed by infection with PR8 virus (MOI = 0.01) for 12, 18, and 24 h. The whole protein lactylation levels were measured by Western blot. **(D-I)** A549 cells were pretreated with oxamate (LDHi) (50 mM) for 1 h, followed by infection with PR8 (D-E), H9N2(F-G), or H3N2 (H-I) virus (MOI = 0.01). Viral NP and PB2 protein levels were measured by Western blot at 24 hpi (D, F, H). Viral titers in the supernatants were determined by $TCID_{50}$ assays (E, G, I). **(J)** Control and SIRT1 knockdown A549 cells were pretreated with oxamate (LDHi) (50 mM) for 1 h. Control cells were infected with H3N2 (MOI = 0.1), and SIRT1 knockdown cells were infected with H3N2 (MOI = 0.03). Control cells with oxamate (LDHi) treatment were infected with H3N2 (MOI = 0.6), while SIRT1 knockdown cells with oxamate (LDHi) treatment were infected with H3N2 (MOI = 0.6) for 24 h. IP assays were performed using anti-NP, anti-PA, or anti-PB2 antibody, with IgG as a negative control, followed by Western blot using anti-pan lac antibody. **(K-M)** Control and SIRT1 knockdown A549 cells were pretreated with oxamate (LDHi) (50 mM) for 1 h, followed by infection with A/PR8/34 (H1N1) (MOI = 0.01) (K), H9N2 (MOI = 0.01) (L), or H3N2 subtype virus (MOI = 0.01) (M) for 24 h. Viral PA and PB2 protein levels were examined by Western blot. **(N)** Control and SIRT1 knockdown A549 cells were infected with lentiviruses expressing control or LDHA shRNA, followed by PR8 virus infection. Viral PA and PB2 protein levels were detected by Western blot. **(O-P)** A549 cells were pretreated with LDHi for 1 h, followed by infection with A/PR8/34 (H1N1) (MOI = 0.01) for 12 h. The cells were then treated with chloroquine (CQ) (20 μM) (O) or MG132 (10 μM) (P) for an additional 10 h respectively. The protein levels of SIRT1 and NP were determined by Western blot. Data are shown as means ± SD (n = 3). *p < 0.05, **p < 0.01, ***p < 0.001.

Table S1

Table S1 Sequences of primers used in this study

Name	Sequence (5' - 3')
SIRT1-F	CGCGGATCCGCCACCATGGCGGACGAGGCGGCCCTCG
SIRT1-R	CGGACTAGTCTATGATTTGTTTGATGGATAGTTCATGTCT
18sRNA-F	AGTTGGTGGAGCGATTTGT
18sRNA-R	TGAGCCAGTCAGTGTAGCG
vRNA-tag-F	GGCCGTCATGGTGGCGAAT
cRNA-tag-F	GCTAGCTTCAGCTAGGCATC
mRNA-tag-F	CCAGATCGTTCGAGTCGT
PR8 NP-vRNA-RT	GGCCGTCATGGTGGCGAATGAATGGACGGAGAACAAGGATTGC
PR8 NP-vRNA-R	CTCAATATGAGTGCAGACCGTGCT
PR8 NP-cRNA-RT	GCTAGCTTCAGCTAGGCATCAGTAGAAACAAGGGTATTTTTCTTT
PR8 NP-mRNA-RT	CCAGATCGTTCGAGTCGTTTTTTTTTTTTTTTTTCTTTAATTGTC
PR8 NP-c/mRNA-R	GCTAGCTTCAGCTAGGCATCAGTAGAAACAAGGGTATTTTTCTTT
SC15-NP-vRNA-RT	GGCCGTCATGGTGGCGAATCGAATGATAAAACGAGGGATTAACGAC
SC15-NP-vRNA-R	TCGCACTTGATCCATCATTGCTCT
SC15-NP-cRNA-RT	GCTAGCTTCAGCTAGGCATCAGTAGAAACAAGGGTATTTTTCTTT
SC15-NP-mRNA-RT	CCAGATCGTTCGAGTCGTTTTTTTTTTTTTTTTTTTTTTTCTTTAATTGTC
SC15-NP-c/mRNA-R	TTCCCTTCGAAAGAGCGACCA
SC15-NA-vRNA-RT	GGCCGTCATGGTGGCGAATTTTGGGCTTAAATATCGGACTCCAC
SC15-NA-vRNA-R	TGCCATGAGTTGACTTCACATAGCG
SC15-NA-cRNA-RT	GCTAGCTTCAGCTAGGCATCAGTAGAAACAAGGGTGTTTTTGTTA
SC15-NA-mRNA-RT	CCAGATCGTTCGAGTCGTTTTTTTTTTTTTTTTTTTTTTTGTTAAATCCTT
SC15-NA-c/mRNA-R	AGACCCAAAGAGAGTAGTGT
shSIRT1#1-F	CCGGCATGAAGTGCCTCAGATATTACTCGAGTAATATCTGAGGCACTTCA TGTTTTTG
shSIRT1#1-R	AATTCAAAAACATGAAGTGCCTCAGATATTACTCGAGTAATATCTGAGGC ACTTCATG
shSIRT1#2-F	CCGGCAGGTCAAGGGATGGTATTTACTCGAGTAAATACCATCCCTTGACC TGTTTTTG
shSIRT1#2-R	AATTCAAAAACAGGTCAAGGGATGGTATTTACTCGAGTAAATACCATCCC TTGACCTG
shLDHA#1-F	CCGGCGGAATAAAGGATGATGTCTTCTCGAGAAGACATCATCCTTTATTC CGTTTTTG
shLDHA#1-R	AATTCAAAAACGGAATAAAGGATGATGTCTTCTCGAGAAGACATCATCC TTTATTCCG
